# Subnanometer localization accuracy in widefield optical microscopy

**DOI:** 10.1038/s41377-018-0031-z

**Published:** 2018-07-11

**Authors:** Craig R. Copeland, Jon Geist, Craig D. McGray, Vladimir A. Aksyuk, J. Alexander Liddle, B. Robert Ilic, Samuel M. Stavis

**Affiliations:** 1 Center for Nanoscale Science and Technology, National Institute of Standards and Technology, Gaithersburg, MD 20899 USA; 2 Maryland NanoCenter, University of Maryland, College Park, MD 20742 USA; 3 Engineering Physics Division, National Institute of Standards and Technology, Gaithersburg, MD 20899 USA

**Keywords:** Imaging and sensing, Super-resolution microscopy, Transmission light microscopy, Wide-field fluorescence microscopy, Optical materials and structures

## Abstract

The common assumption that precision is the limit of accuracy in localization microscopy and the typical absence of comprehensive calibration of optical microscopes lead to a widespread issue—overconfidence in measurement results with nanoscale statistical uncertainties that can be invalid due to microscale systematic errors. In this article, we report a comprehensive solution to this underappreciated problem. We develop arrays of subresolution apertures into the first reference materials that enable localization errors approaching the atomic scale across a submillimeter field. We present novel methods for calibrating our microscope system using aperture arrays and develop aberration corrections that reach the precision limit of our reference materials. We correct and register localization data from multiple colors and test different sources of light emission with equal accuracy, indicating the general applicability of our reference materials and calibration methods. In a first application of our new measurement capability, we introduce the concept of critical-dimension localization microscopy, facilitating tests of nanofabrication processes and quality control of aperture arrays. In a second application, we apply these stable reference materials to answer open questions about the apparent instability of fluorescent nanoparticles that commonly serve as fiducial markers. Our study establishes a foundation for subnanometer localization accuracy in widefield optical microscopy.

## Localization microscopy: approaching atomic accuracy across a wide field

An innovative approach to calibrating optical microscopes greatly improves the accuracy of position measurements of single molecules and nanoparticles emitting light. Researchers at the National Institute of Standards and Technology (NIST) and the University of Maryland, in Maryland, USA, have developed a reference material and calibration method that reduces errors in localization microscopy to within a few atomic diameters across a field of hundreds of micrometers. The method uses a nanofabricated array of apertures, or holes, to transmit light and form a standard image that enables correction of localization data. This approach eliminates systematic errors, which vary across the field, and improves the localization accuracy by up to a factor of 10,000. Their work advances the ability to perform quantitative measurements by localization microscopy in diverse disciplines such as cell biology, micromechanics, and nanotechnology.

## Introduction

**Fig. 1:**
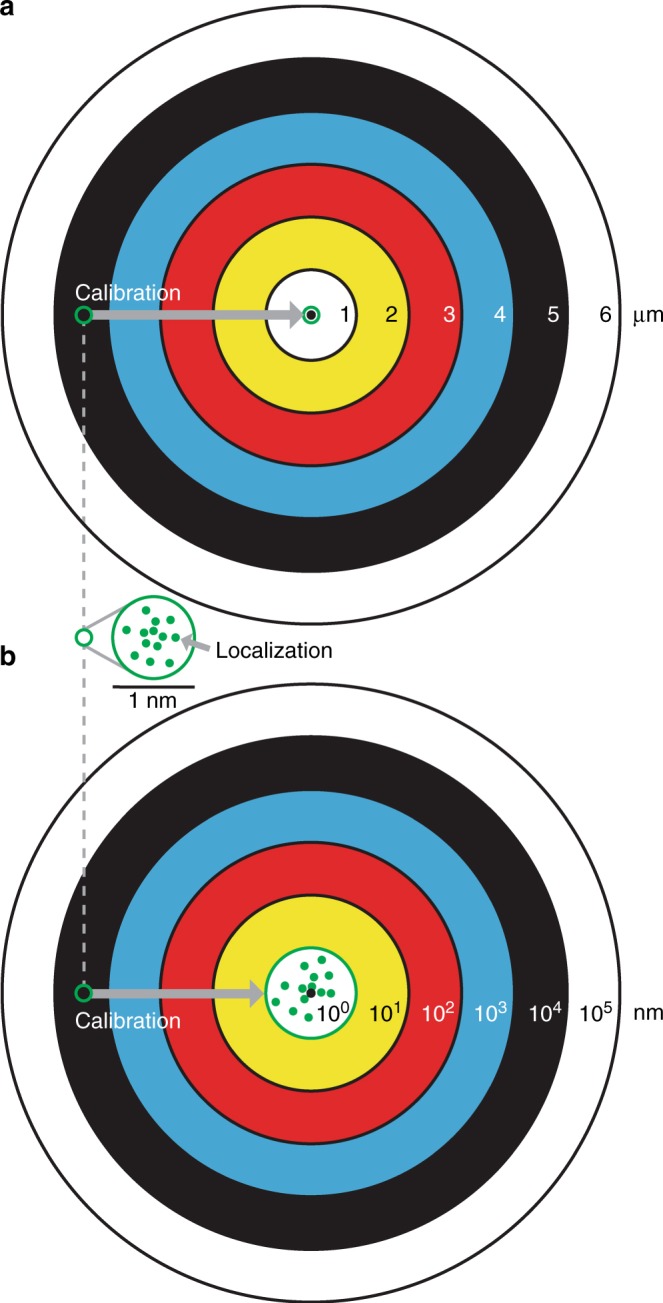
Precision and accuracy in localization microscopy. **a** Schematic showing a linear target. **b** Schematic showing a logarithmic target. Green dots are localization data. Their scatter indicates statistical uncertainty at the subnanometer scale, which is not apparent on the linear target as systematic errors can be four orders of magnitude larger. This discrepancy requires a logarithmic target to illustrate both precision and accuracy. Calibration of the measurement system and correction of localization data ensures that precision is the limit of accuracy^13^

Optical microscopy methods of localizing small emitters are broadly useful in such fields as cell biology, nanoscale fabrication, cryogenic physics, and microelectromechanical systems^1^. Both precision^2–4^ and accuracy are fundamental to localization microscopy^5,6^. Localization of single fluorophores with a statistical uncertainty of tens of nanometers is common, and subnanometer uncertainty is possible for fluorophores^7^ and readily achievable for brighter emitters such as particles^8^. Whereas improving localization precision generally requires counting more signal photons by increasing the intensity and stability of emission^9,10^, achieving commensurate localization accuracy presents diverse challenges in the calibration of an optical microscope as a nonideal measurement system. Such calibration involves not only the discrete parts of the system but also the interaction of those parts during a measurement and is rarely, if ever, implemented. This can cause overconfidence in measurement results with statistical uncertainties at the nanometer scale that are invalid due to larger systematic errors. These errors can extend into the micrometer scale when localizing emitters across a wide field, as is often necessary for imaging microstructures and tracking motion^11,12^. The discrepancy between precision and accuracy can be so large as to require a logarithmic target to illustrate, as Fig. 1 shows.

The root cause of the problem is a lack of reference materials and calibration methods that are optimal for localization microscopy, analogous to those for optical imaging at larger scales^14^. Small particles are useful for mapping certain effects of optical aberrations^15–17^. However, their size distribution and random deposition can result in nonuniform sampling of the imaging field, fluorophores in particles often have a different emission spectrum from that of fluorophores in solution, and evaluating magnification^18^ requires a specification of distance between emitters. DNA origami can control the submicrometer distance between a few fluorophores^19,20^, but this approach has limitations of emitter intensity and stability, as well as sampling uniformity. Stages require their own calibration to scan emitters through the imaging field, while microscope instability can limit sampling accuracy^21–23^. Arrays of subresolution apertures enable calibration of both aberrations and magnification, with intense and stable emission, and uniform and accurate sampling^24^. Recent studies have used aperture arrays to calibrate the effects of chromatic aberrations on image registration^22,23,25,26^, sample orientation and aberrations in three dimensions,^27^ and image pixel size^28^. However, these studies have not quantified the critical dimensions of an aperture array to produce a reference material, demonstrated all functions of an aperture array for microscope calibration, or reached the performance limits of the corresponding calibration methods. Other factors contribute to the overall problem, as follows.

Electron-multiplying charge-coupled-device (EMCCD) cameras were common at the advent of localization microscopy and their calibration continues^29^. Complementary metal-oxide-semiconductor (CMOS) cameras are of increasing interest due to advantages of performance and cost but have nonuniform sensitivity and read noise. Initial studies tested the effects of CMOS noise on localization^30^ and improved the localization of single fluorophores^31,32^. However, no study has calibrated over the full dynamic range of a CMOS camera to maximize the number of signal photons and minimize statistical uncertainty. Previous studies have improved illumination uniformity^33^ and performed flatfield corrections but have not accounted for all related CMOS nonuniformities.

Localization analysis extracts information from optical images. Maximum-likelihood and weighted least-squares algorithms^34,35^, with specific estimators for CMOS cameras^31,32^, compete on the basis of accuracy and efficiency. However, previous studies have not evaluated the performance of each algorithm in the presence of discrepancies between model approximations of the point spread function and experimental data. The resulting fitting errors are common for models that neglect deformations from aberrations^36–38^, which vary across a wide field.

Finally, localization of a fiducial marker such as a small particle often provides a reference position for correcting systematic errors from unintentional motion of the sample or microscope^9,39–41^. A typical but critical assumption is that the fiducial is motionless with respect to the sample. However, there are open questions about whether nanoparticle fiducials are truly static on imaging substrates^15,34,39^. Confounding this issue, microscope systems are not perfectly stable, and there is no appropriate reference material for assessing their subnanometer stability across a wide field.

In this study, we present a comprehensive solution to this overall problem, reducing localization errors from a widefield optical microscope by up to four orders of magnitude and transforming the microscope into a quantitative metrology system. We develop aperture arrays into prototype reference materials with multiple functions and combine them with novel methods to calibrate the parts of the system and their interaction during a measurement. We validate our widefield measurements and quantify localization error approaching the scale of atomic diameters across a submillimeter field, for multiple colors and emission sources. We apply our new measurement capability to introduce the concept of critical-dimension localization microscopy of aperture arrays and to answer open questions about the apparent motion of nanoparticle fiducials. By minimizing and quantifying systematic errors at subnanometer scales, we enable rigorous confidence in precision as the limit of accuracy for localization microscopy.

## Materials and methods

### Aperture arrays

We design^42^ and fabricate square arrays of circular apertures with nominal diameters ranging from 200 nm to 500 nm in titanium and platinum films with a total thickness of approximately 100 nm on silica substrates with a thickness of approximately 170 µm. We use two different electron-beam lithography systems to pattern independent arrays and test the accuracy of aperture placement. Both lithography systems have traceable laser interferometers that measure stage position with a resolution of approximately 0.6 nm in the *x* and *y* directions to calibrate electron-beam position and to confirm the absence of, or correct for, electron-optical aberrations. To avoid additional errors of aperture placement from stage motion of the lithography systems, we limit the lateral extents of our arrays to single write fields. Further details are in Supplementary Notes S1 and 2, Supplementary Table S1, and Supplementary Figs. S1–4. To develop our calibration methods, we initially assume placement accuracy and we assume that random errors determine placement precision, as we define in Supplementary Table S2. We subsequently measure these dimensional properties.

### Fluorescent samples

For some measurements, we fill the aperture array with a solution of boron-dipyrromethene dye at a concentration of approximately 200 μM in N,N-dimethylformamide. We also test fluorescent nanoparticles as fiducial markers. The manufacturer specifies polystyrene spheres with a mean diameter of 220 nm, containing boron-dipyrromethene dye molecules and having a carboxylic acid coating. We disperse the nanoparticles in pure water, deposit 10 µL of the suspension onto a borosilicate coverslip with a thickness of approximately 170 µm and a poly-D-lysine coating, and remove the suspension after 1 min. We expect the nanoparticles to bind electrostatically to the coverslip. We cover the sample surface with pure water and seal it with another borosilicate coverslip for imaging. The emission spectra of the fluorescent dyes in solution and in nanoparticles are in Supplementary Fig. S6.

### Optical microscope

Our microscope has an inverted stand, a scanning stage that translates in the *x* and *y* directions with a sample holder that rotates around these axes, and a piezoelectric actuator that translates an objective lens in the *z* direction with a nominal resolution of 10 nm. We typically use an objective lens with a nominal magnification of 63×, a numerical aperture of 1.2, and an immersion medium with an index of refraction of 1.33, resulting in a nominal depth of field of 0.95 µm at a wavelength of 500 nm. We reconfigure the microscope to epi-illuminate fluorescent dye in aperture arrays and fluorescent nanoparticles on a microscope coverslip or transilluminate empty aperture arrays with a light-emitting diode (LED) array. The numerical aperture of the transilluminator condenser is 0.55. The emission spectra for the three LED arrays that we use are in Supplementary Fig. S6. The microscope has a CMOS camera with 2048 pixels by 2 048 pixels, each with an on-chip size of 6.5 μm by 6.5 µm. We always operate the camera with water cooling and without on-board correction of pixel noise. We typically operate the camera in fast-scan mode, cool the sensor to −10 °C, and calibrate the imaging system for these parameters. In tests of fiducial stability, we operate the camera in slow-scan mode and cool the sensor to −30 °C. For fluorescence imaging, we use an excitation filter with a bandwidth from 450 nm to 500 nm, a dichroic mirror with a transition at 505 nm, and an emission filter with a bandwidth from 515 nm to 565 nm. We always equilibrate the microscope for at least 1 h before acquiring data at an ambient temperature of approximately 20 °C. Representative micrographs of an aperture array and nanoparticle fiducials are in Supplementary Figs. S3–5.

### Sample orientation and position

We level the aperture array by iteratively rotating it around its *x* and y axes, and translating the objective lens in the *z* direction to simultaneously focus on apertures at the four corners of the imaging field. We test an alternate method for leveling the sample by analysis of Zernike coefficients, as Supplementary Note S3 describes. A schematic of our sample holder and corresponding results are in Supplementary Fig. S7. For all measurements, unless we note otherwise, we translate the objective lens through *z* to obtain a series of images around optimal focus for each aperture in an array, as Supplementary Note S4 describes and Supplementary Fig. S8 shows. We image at array centers unless we note otherwise.

### Camera calibration

For each pixel *i*, we measure pixel value offset $o_i$ as the mean and read noise $\sigma _{{\mathrm{read}},\,i}^2$ as the variance of 60 000 images^31^ with the camera shutter closed. We determine flatfield corrections by imaging a white, planar object that is far out of focus and effectively featureless, at nine illumination levels spanning the dynamic range of the imaging sensor, $FF_i = \frac{{\overline {I_i^ \ast } - o_i}}{{\bar I}}$, where $\overline {I_{\mathrm{i}}^ \ast}$ is the mean value before calibration of pixel $i$ from 15 000 images at an illumination level, $o_i$ is the pixel value offset, and $\bar I$ is the mean value of $\overline {I_i^ \ast } - o_i$ from all pixels. The total noise of each pixel is the variance of the pixel value before calibration minus the pixel value offset from the 15 000 images at each illumination level. Plots and histograms of pixel value offset and read noise are in Supplementary Fig. S9.

### Model fitting

We fit polynomial models to data using unweighted least-squares estimation and the Levenberg–Marquardt algorithm to determine optimal focus, characterize CMOS response, and calculate Zernike coefficients. We fit Gaussian models to images of point spread functions using various estimators and the Nelder–Mead simplex algorithm^43^ to localize single emitters.

## Results and discussion

### Terminology

For processes ranging from aperture fabrication to data registration, we define qualitative terms, sources of error, and corresponding quantities in Supplementary Table S2. Our terminology is consistent with both common use and a common guide for metrology vocabulary^13^.

### Aperture array

**Fig. 2:**
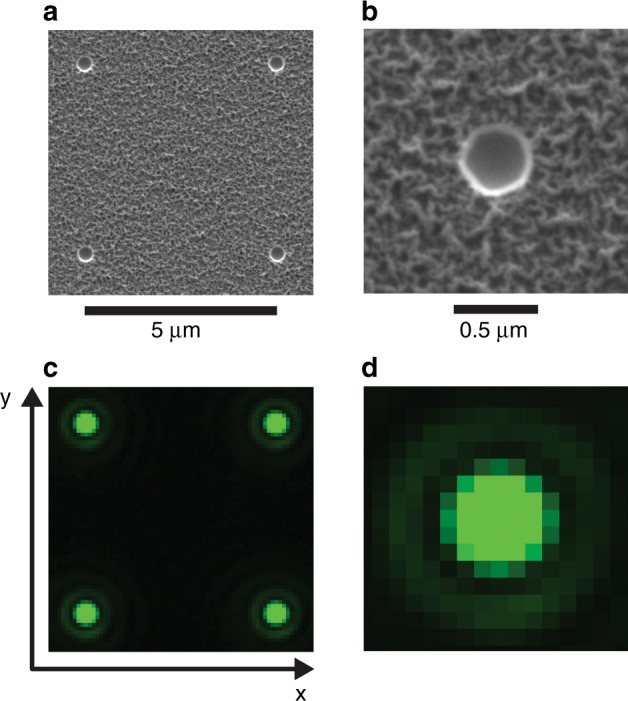
Aperture array. **a–b** Scanning electron micrographs showing representative apertures in a metal bilayer on a silica substrate. **a** The array has a nominal pitch of 5 µm. **b** Apertures have nominal diameters of 400 nm and smaller functional diameters, as Supplementary Note S7 and Supplementary Table S1 describe. **c–d** Brightfield optical micrographs showing representative apertures transmitting light. False color represents the peak illumination wavelength of 500 nm. **c** Four apertures form unit cells for pitch analysis. **d** The image of an aperture closely resembles the point spread function of the imaging system

We test epi-illumination of a fluorescent dye in the apertures^27^ and transillumination of empty apertures^23^ as relevant configurations for localization microscopy. Whereas the dye solution degrades and requires cleaning, empty apertures are more stable and thus appropriate for developing our calibration methods. After doing so, we revisit the difference between the two configurations. Transillumination of empty apertures produces an array of point sources, as Fig. 2 and Supplementary Fig. S3 show, and as Supplementary Table S1 and Supplementary Note S7 describe. An array pitch of at least 5 µm ensures that the point spread functions from adjacent apertures do not overlap significantly, as Supplementary Fig. S4 shows.

### CMOS calibration

Accurate localization of aperture images first requires calibration of our CMOS camera, which we find is even less uniform than indicated by previous studies. Nonuniform pixel gain, sensor packaging, and illumination intensity cause significant variation in pixel value, motivating a flatfield correction. This correction increases with pixel value mean through the bottom 5% of the dynamic range and then remains nearly constant over the remaining 95%, as Supplementary Fig. S10a–b shows. A recent study did not identify this trend but presented localization algorithms that still achieved the Cramér–Rao lower bound^32^. Therefore, we use the constant correction in our analysis of pixel values that span the full dynamic range. Total noise, or pixel value variance, including read noise, shot noise, and fixed-pattern noise, does not depend linearly on pixel value mean over the full dynamic range, as Supplementary Fig. S10c–d shows, in contrast to a linear approximation from Poisson statistics at low pixel values. A quartic polynomial is a better approximation, but the linear approximation results in localization that is equally accurate and more efficient. Further details are in Supplementary Note S5 and Supplementary Table S3.

### Localization algorithm

Aberrations, such as from objective lenses^44^, can become significant across a wide field and deform the point spread function in ways that are typically unpredictable. Most localization algorithms do not account for such deformation, and one even requires its absence^45^. Previous studies have not fully explored the effects of fitting errors^35,46^ on the performance of weighted least-squares^32^ or maximum-likelihood^31^ estimation. These algorithms can include information from CMOS calibration and shot noise, unlike unweighted least-squares. There are arguments for and against each algorithm^32,34^. Rather than strictly adhering to one algorithm or another, we use the aperture array to test their performance in the presence of fitting errors from aberration effects, which vary across a wide field. For this test, we select a bivariate Gaussian approximation of the point spread function,

1 $$	{G}_{\mathrm{biv}}\left({x,y}\right)=A {\cdot}{\mathrm{exp}} \\ 	-{\left(\frac{1}{2(1-{\rho}^2)}\left[\frac{{({x}-{x}_{0})}^2}{\sigma_{x}^2}-2{\rho}\frac{({x}-{x}_{0})({y-y}_{0})}{\sigma_{x}\sigma_{y}}+\frac{{({y-y}_{0})}^2}{\sigma_{y}^2}\right]\right)+{C}}$$

where $A$ is the amplitude, $x_0$ is the position of the peak in the *x* direction, $y_0$ is the position of the peak in the *y* direction, $\sigma_x$ is the standard deviation in the *x* direction, $\sigma_y$ is the standard deviation in the *y* direction, $\rho$ is the correlation coefficient between the *x* and *y* directions, and $C$ is a constant background. Unlike a univariate Gaussian function, this model has some empirical ability to accommodate asymmetry from deformation of the point spread function^24,47^, which can be significant, as Fig. 3 shows at a corner of the imaging field, 140 µm away from its center.

**Fig. 3:**
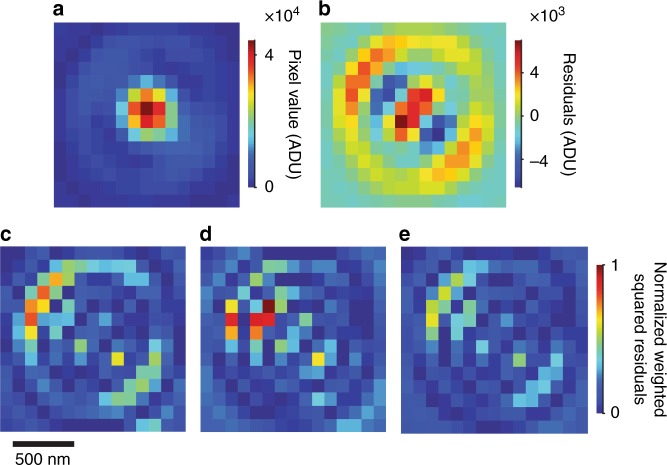
Localization algorithms. **a** Brightfield optical micrograph showing the localization region of interest containing a point spread function with asymmetry from aberrations. Pixel values are in analog-to-digital units (ADU). False color enhances contrast. We fit a bivariate Gaussian model to the data to test the estimation performance of three localization algorithms in the presence of model discrepancy. **b** Plot showing residuals from a fit using the light-weighting objective function. **c–e** Plots showing weighted squared residuals on a normalized scale. **c** Weighted least-squares heavily weights the first Airy ring. **d** Maximum-likelihood heavily weights between the central peak and Airy ring. **e** Light-weighting results in more uniform weighting than either (**c**) or (**d**) and improves empirical localization precision on average

In light of the fitting errors that result, we introduce an empirical objective function for robust parameter estimation. The light-weighting objective function reduces the effect of fitting errors whether the model overestimates or underestimates the data,

2 $${\hat{\mathrm{\Theta }}} = {\mathrm{argmin}}\left[ {\mathop {\sum }\limits_{i} \frac{{\left( {{I}_{i} - {E}_{i}} \right)^2}}{g \cdot {\mathrm{max}}\left( {{I}_{i},{E}_{i}} \right) + \sigma _{{\mathrm{read}},{i}}^{2}}} \right]$$

where ${\hat{\mathrm{\Theta }}}$ is the estimate for the parameter set ${\hat{\mathrm{\Theta }}} = \left\{ {A,\sigma _x,\sigma _y,\rho ,x_0,y_0,C} \right\}$, $i$ indexes each pixel, $I_i$ is the experimental pixel value after CMOS calibration, $E_i$ is the expected or model pixel value, $g$ is the nominal gain of the camera, and $\sigma _{{\mathrm{read}},\,i}^2$ is the pixel read noise. The use of max$\left( {{I}_{i},{E}_{i}} \right)$ selects either weighted least-squares ($I_i$ > $E_i$) or maximum-likelihood ($I_i$ < $E_i$) to reduce the weights of pixels with large residuals due to model discrepancy. Further details are in Supplementary Note S6.

The algorithm performance depends on both the deformation extent and the photon count, as Supplementary Fig. S11 and Supplementary Table S4 show. For our wide field and intense emitters, light-weighting improves empirical localization precision on average, as Supplementary Table S4 shows. In field regions with large deformation, unweighted least-squares improves localization precision relative to the other algorithms. In field regions with small deformation, light-weighting, maximum-likelihood, and weighted least-squares perform comparably. The same is true in the case that the localization region of interest excludes regions of the point spread function that cause the largest fitting errors, but doing so degrades empirical localization precision on average, as Supplementary Table S4 shows. We subsequently quantify localization error, including any effects of fitting errors.

### Aberration effects

**Fig. 4:**
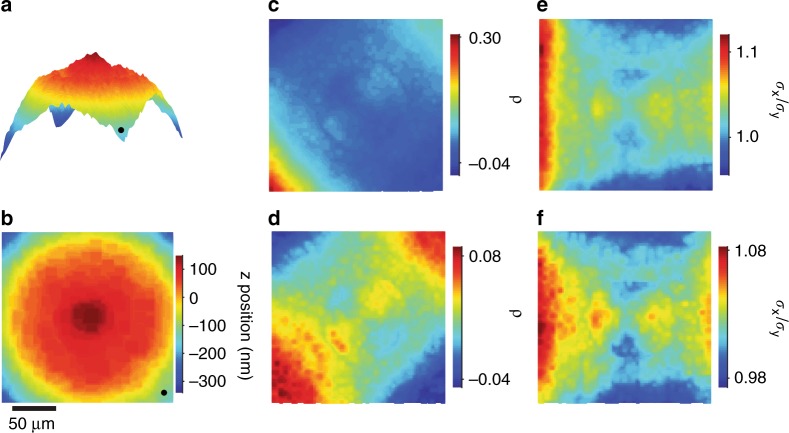
Field curvature and point spread function deformation. **a**–**b** Plots showing the curving field of the imaging system. Black dots mark the same corner. The optimal focal plane is at z = 0 nm. **c** Plot showing a larger range of $\rho$ from a single image at the optimal focal plane, maximizing the mean amplitude of all point spread functions. **d** Plot showing a smaller range of $\rho$ from multiple images along the curving field, maximizing the amplitude of each point spread function. **e** Plot showing $\sigma _x/\sigma _y$ from a single image at the optimal focal plane. **f** Plot showing $\sigma _x/\sigma _y$ from multiple images along the curving field. For these plots and subsequent plots showing optical effects, we use linear interpolations of data between aperture positions

Aberrations degrade localization accuracy through several effects. In our experimental system, a silica substrate of standard thickness and high quality underpins the aperture array and is therefore part of the microscope system and its calibration. Additional calibration may be necessary for aberration effects from an experimental sample^48^. We begin to calibrate aberration effects by characterizing the bivariate Gaussian approximation of the point spread function in three dimensions. We image the aperture array through focus, and locate optimal focus for each aperture as the *z* position that maximizes the amplitude of the resulting point spread function, as Supplementary Fig. S8 shows. The field curves in the *z* direction over a range of approximately  500 nm, as Fig. 4a–b show. We confirm the effective flatness of the aperture array, as Supplementary Fig. S2 shows. Without such characterization, a nonplanar array can corrupt calibration for localization in three dimensions^27^. The complex curvature of the field motivates the use of an aperture array to uniformly sample it, and has several consequences. Not all objects across the field can be at optimal focus simultaneously. Many experiments permit acquisition of only a single micrograph, which can be at a *z* position that maximizes the mean amplitude of point spread functions across the field. We define this optimal focal plane as *z* = 0 nm in Fig. 4b. If the quasistatic imaging of stable emitters is feasible, then acquiring multiple micrographs along the curving field allows for optimal focus of each point spread function.

For the bivariate Gaussian approximation of the point spread function, the dimensionless parameters $\rho$ and $\sigma _x/\sigma _y$ describe asymmetries resulting from deformation. We extract these parameters from one image at the optimal focal plane, as Fig. 4c, e show, and from multiple images along the curving field at which all apertures are in optimal focus, as Fig. 4d, f show. In either case, the parameters have a similar field dependence. Imaging through focus reduces the range of $\rho$ by a factor of approximately three but has little effect on $\sigma _x/\sigma _y$. Either analysis can improve localization by fixing or improving initial guesses of model parameters in minimization algorithms, which can be important for localization accuracy^35^. These results also imply the potential for parameterizing accurate models of the point spread function, as well as for exploiting intrinsic aberrations to localize emitters in three dimensions.

**Fig. 5:**
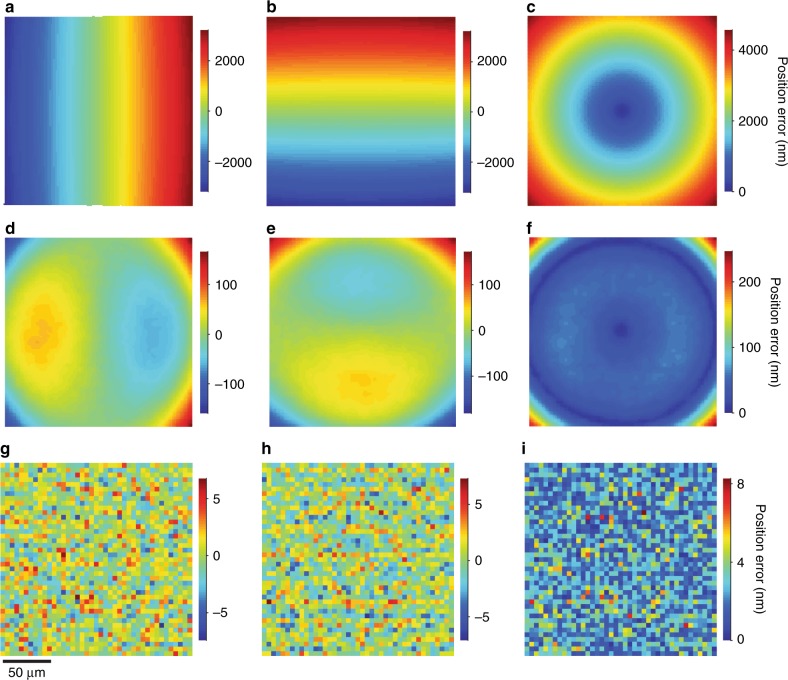
Position errors. **a–c** Plots showing position errors in (**a**) the *x* direction, (**b**) the *y* direction, and (**c**) total magnitude, due mostly to using the nominal value of image pixel size of 103 nm. **d**–**f** Plots showing position errors in (**d**) the *x* direction, (**e**) the *y* direction, and (**f**) total magnitude, after applying a similarity transformation to map the data in (**a**–**c**) to an ideal array, due mostly to using the mean value of image pixel size of 99.94 nm. **g–i** Plots showing position errors in (**g**) the *x* direction, (**h**) the *y* direction, and (**i**) total magnitude, after applying a correction model to the localization data in (**d**–**f**), due mostly to placement precision

From one micrograph at the optimal focal plane, we localize each aperture and perform a similarity transformation to map an ideal array, with a pitch that is identical to the nominal value of 5 μm, to the localization data. This transformation consists of planar translation and rotation, and uniform scaling to determine the mean value of image pixel size. The differences between the positions that we measure and the nominal positions in the ideal array define position errors. The transformation scale factor results in a mean value of image pixel size of 99.94 nm, which is 3% smaller than the nominal value of 103 nm. We revisit the uncertainty of image pixel size. Using the nominal value of image pixel size, which is a common but inadvisable practice, results in position errors of up to 4.5 µm, as Fig. 5a–c show. Using the mean value of image pixel size resulting from the similarity transformation reduces these position errors by a factor of more than 18, however, the errors are still as large as 250 nm and vary nonmonotonically across the field, as Fig. 5d–f show. These position errors are due primarily to pincushion distortion but also to field curvature and deformation of the point spread function. This extent of magnification calibration is comparable to that of a previous study that averaged over these effects in determining a mean value of image pixel size^18^, and demonstrates the utility of sampling the field with an aperture array to further reduce systematic errors from aberration effects.

With other objective lenses, our microscope system shows comparable aberration effects of variable magnitude and field dependence, as Supplementary Fig. S12 and Supplementary Table S5 show. All of the objective lenses that we test result in mean values of image pixel size that are smaller than the nominal values by approximately 3%, indicating that our microscope tube lens is the primary source of this systematic error. This finding is consistent with our observations of other microscope systems from the same manufacturer, which we do not show. The lens with the lowest numerical aperture results in the smallest position errors, revealing an unnecessary competition between collection efficiency and magnification uniformity that exists in the absence of calibration.

### Error correction

**Fig. 6:**
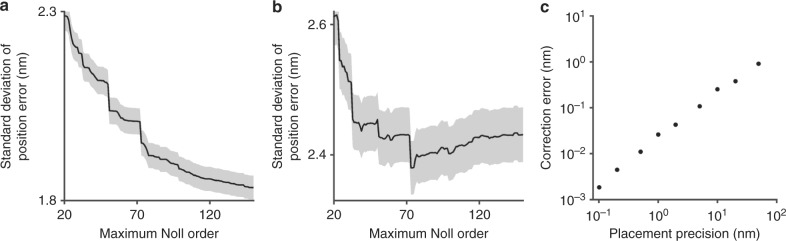
Correction model. **a**–**b** Plots showing representative values of the standard deviation of position errors in a single lateral dimension after correction, as a function of the number of consecutive Zernike polynomials in the model, or the maximum Noll order. A maximum Noll order of less than 20 corrects the largest fraction of the position errors. **a** At the center of the standard array from which we derive the model, the standard deviation decreases monotonically with maximum Noll order as the model corrects position errors due primarily to aberrations. **b** After applying the model from (**a**) to a different region of the standard array, the standard deviation decreases to a minimum at a maximum Noll order of 73 and then increases with additional orders, indicating erroneous inclusion of position errors due to placement precision at the array center. Plots for other regions of the array are similar. Gray bounds are one standard error. **c** Plot showing correction error, which increases approximately linearly with placement precision. Standard errors are smaller than the data markers

We model the position errors in Fig. 5d–f by a linear combination of consecutive Zernike polynomials^49^ to develop a widefield correction that is applicable to position data from many forms of localization microscopy. The correction takes as input the inaccurate position of an emitter from a localization measurement, and gives as output its accurate position. The similarity transformation gives the value of image pixel size. At the center of the standard array from which we derive the model, the standard deviation of position error decreases monotonically with maximum Noll order, as Fig. 6a shows. Sharp decreases correspond to polynomials with odd radial degrees greater than 1 and azimuthal degrees of 1 and −1, providing insight for optimization of the model by selection of a subset of nonconsecutive Zernike polynomials.

We quantify the effect of placement precision on the correction model by two novel tests. First, we apply the correction to a different region of the standard array. The standard deviation of position error decreases to a minimum at a maximum Noll order of 73 and then increases, as Fig. 6b shows. This trend indicates a limit beyond which additional consecutive Zernike polynomials erroneously correct position errors due to placement precision at the array center, degrading correction accuracy. To test this effect in the correction model of maximum Noll order 73, we simulate position errors due to placement precision as the standard deviation of a normal distribution around a mean pitch of 5 µm, and apply the correction to the resulting positions. The correction error depends approximately linearly on the magnitude of placement precision, as Fig. 6c shows, and contributes less than 0.05 nm to the localization error for our aperture array.

The correction model of maximum Noll order 73 reduces the position errors in Fig. 5d–f by another factor of 30, resulting in position errors in the *x* and *y* directions that are apparently random, as Fig. 5g–i show. The mean value of position errors is zero by definition of the similarity transformation, and the standard deviations of position errors for this standard array are in Table 1. We revisit these quantities to clarify their meaning.

**Table 1:** Standard deviation of position errors from widefield measurements

**Array**	***x*** **direction (nm)**	***y*** **direction (nm)**
Standard process	1.95 ± 0.03	1.97 ± 0.03
Low current, long dwell	2.43 ± 0.04	2.00 ± 0.03
Low current, many passes	2.11 ± 0.04	1.35 ± 0.02

Uncertainties are one standard error of the standard deviation^50^

### *Z* position

Optimal use of the aperture array requires control of its *z* position with respect to the imaging system, and, by extension, its orientation around the *x* and *y* axes^51^. Although our nominal depth of field of nearly 1 µm is much greater than our positioning resolution in the *z* direction of 10 nm, position errors in the *x* and *y* directions are still sensitive to changes in the *z* direction that are as small as 10 nm, which deform the imaging field radially, as Supplementary Figs. S13 and S14 show. For *z* positions beyond 150 nm from optimal focus, the standard deviation of position errors increases by more than 1 nm. Correction of experimental data will typically require disengagement of a reference material and engagement of an experimental sample, which can cause localization errors from variation in *z* position. This sensitivity also indicates the importance of microscope stability, as we investigate subsequently.

### Scanning measurements

To validate our widefield measurements and correction of position errors, we scan the aperture array to sequentially position all apertures that comprise the data in Fig. 5 within the central 100 µm^2^, or 0.2%, of the imaging field area. This scanning measurement minimizes the effects of photon-optical aberrations to the extent that we can sample them with an array pitch of 5 µm, as Figs. 4 and 5d–f show. Pitch values within unit cells of the array are independent of the resolution and repeatability of the scanning stage of the optical microscope. For 1 600 pairs of apertures, scanning measurements result in pitch values that are apparently consistent with widefield measurements, as Supplementary Table S6 shows.

This consistency is only superficial, however, as a deeper analysis shows that scanning and widefield measurements each include multiple sources of error and enables discrimination between the errors. Further details are in Supplementary Note S8. From this analysis, we determine that placement precision results in position errors with a standard deviation of 1.71 nm ± 0.05 nm in the *x* direction and 1.81 nm ± 0.05 nm in the *y* direction^52^, and that widefield measurements have a localization error of 0.62 nm ± 0.20 nm in the *x* direction and 0.72 nm ± 0.19 nm in the *y* direction, independently of empirical localization precision. These uncertainties are standard errors. Further details are in Supplementary Table S7.

Virtually all measurements have errors that limit accuracy at some scale, and our quantification of localization error in widefield measurements is an important advance. One metric for assessing the resulting performance is the field size to localization error ratio of 3 × 10^5^. To our knowledge, this is the best accuracy for a localization measurement in widefield optical microscopy.

### Chromatic aberrations

Registration of localization data from different wavelengths can result in errors from chromatic aberrations. To study these effects, we sequentially transilluminate the aperture array with three colors, acquiring three micrographs at each *z* position. For each color, we determine the *z* position of the optimal focal plane, the mean value of image pixel size, and the correction model. The mean values of image pixel size differ due to lateral chromatic aberration, and the *z* positions of the optimal focal planes differ due to axial chromatic aberration, as Supplementary Table S8 shows.

**Fig. 7:**
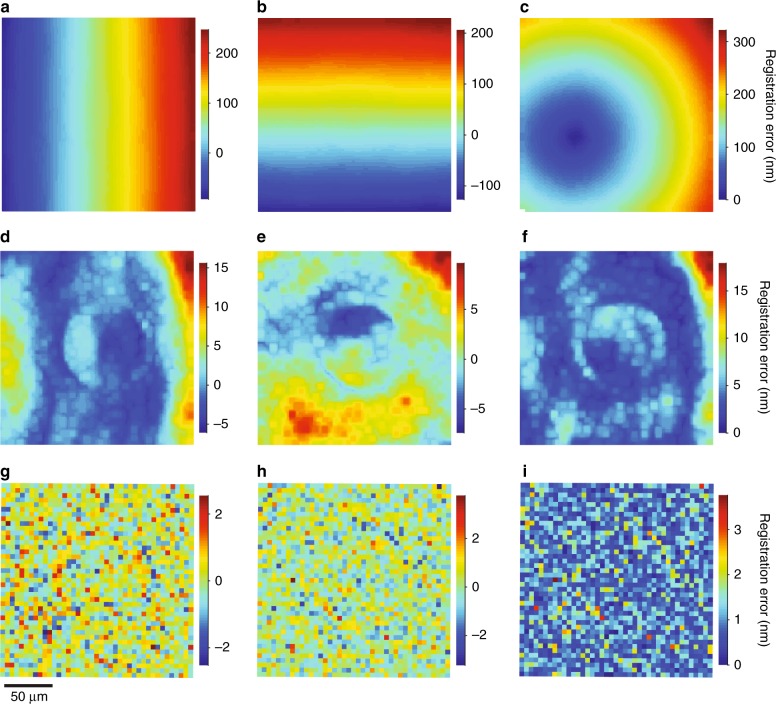
Registration errors. **a**–**c** Plots showing registration errors in (**a**) the *x* direction, (**b**) the *y* direction, and (**c**) total magnitude, due mostly to different mean values of image pixel size and a lateral offset for localization data of different colors. **d–f** Plots showing registration errors in (**d**) the *x* direction, (**e**) the *y* direction, and (**f**) total magnitude, after applying a similarity transformation to the localization data, due mostly to variable distortion from chromatic aberration. **g–i** Plots showing registration errors in (**g**) the *x* direction, (**h**) the *y* direction, and (**i**) total magnitude, after applying correction models to the localization data before a similarity transformation, due mostly to localization error and empirical localization precision

The difference in mean values of image pixel size, and a lateral offset, dominate registration errors, as Fig. 7a–c shows for peak wavelengths of 500 nm and 630 nm. We reduce the effects of axial chromatic aberration by selecting and registering micrographs at the optimal focal plane for each color. Registration errors increase for a common *z* position for multiple colors due to defocus of at least one color, as Supplementary Fig. S15 shows. A similarity transform of the localization data before registration reduces the errors in Fig. 7a–c, resulting in systematic errors from the dependence of distortion on color, extending to over 15 nm, as Fig. 7d–f shows. Previous studies have empirically modeled such errors without characterizing the contributing effects^22,23,25,26^. These errors are due only to chromatic aberrations, adding to the errors in Fig. 5. In a novel analysis, we correct the data from each color prior to the similarity transform. This correction removes the systematic errors from Fig. 5a–f and Fig. 7d–f, resulting in registration errors that are apparently random, as Fig. 7g–i shows. The corresponding localization errors are 0.35 nm ± 0.01 nm in the *x* direction and 0.47 nm ± 0.01 nm in the *y* direction. These uncertainties are standard errors. These localization errors are consistent with but smaller than the localization error that we determine from a comparison of widefield and scanning measurements, indicating the existence of systematic components of localization error that cancel in data registration. Further details and the registration of other colors are in Supplementary Fig. S16, Supplementary Note S9, and Supplementary Tables S9 and 10.

### Emission source

We compare transillumination of empty apertures^23^ and epi-illumination of fluorescent dye in the apertures^27^. The emission wavelengths are similar but not identical for this comparison, as Supplementary Fig. S6 shows. As an exemplary quantity for comparison, the mean values of image pixel size are 100.07 nm for transillumination and 100.16 nm for epi-illumination, which differ by more than is attributable to any potential effects of chromatic aberrations, as Supplementary Table S8 shows. These results indicate effects of the illumination and aperture optics, and the requirement for matching the emission of light from apertures to an experimental system to calibrate it. Our reference material and calibration method work equally well for either experimental configuration, indicating their general applicability, as Supplementary Fig. S17 shows. Diverse sample environments are relevant to localization microscopy, motivating future studies of their effects on fluorescence emission and microscope calibration.

### Critical dimensions

We have assumed the absence of effects of electron-optical aberrations on placement accuracy, which would corrupt calibration of systematic effects of photon-optical aberrations. We test this possibility in two ways. First, because the lateral extent of the aperture array exceeds that of the imaging field, we can independently measure different regions of the array. If electron-optical aberrations were significant, then the photon-optical correction would erroneously include their effects at the array center, resulting in systematic errors upon application of the correction to other regions. No such errors are apparent, as Supplementary Fig. S14 shows. Second, we sample the full extent of the aperture array by scanning 100 pairs of apertures through the central 0.2% of the imaging field area. No systematic variation in pitch from electron-optical aberrations is apparent, as Supplementary Fig. S18 shows.

In a novel test of placement accuracy, we pattern an independent aperture array using a second lithography system. Widefield measurements reveal that the two arrays differ in mean pitch by 0.01 pixels or approximately 1 nm, as Supplementary Table S11 shows. This difference is extremely statistically significant, with a *p* value of 0.0006 for the *x* direction and 0.0004 for the *y* direction, but exceeds the position resolution of the lithography stages by less than a factor of two and is approximately half of the standard deviation of position errors due to placement precision. This analysis provides an estimate of placement accuracy, with a corresponding systematic error of image pixel size of 1 nm/5 000 nm = 0.02%. Importantly, such errors sum arithmetically with distance, as Fig. 5a–f shows, so that placement accuracy ultimately limits localization accuracy^28^. However, this limitation of the reference material results in a relative error of only 0.02% in our analysis of placement precision and empirical localization precision. To our knowledge, this is the most rigorous analysis of a reference material for localization measurements across a wide field.

**Fig. 8:**
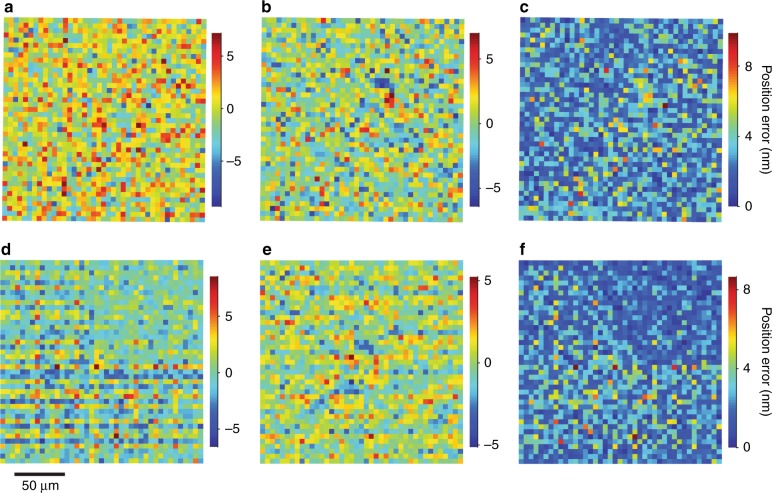
Patterning processes. **a-c** Plots showing position errors in (**a**) the *x* direction, (**b**) the *y* direction, and (**c**) total magnitude, after correcting measurements of aperture positions from an array that we pattern by decreasing the electron-beam current from 1.0 nA to 0.2 nA and increasing the dwell time proportionately to deliver the same dose. **d–f** Plots showing position errors in (**d**) the *x* direction, (**e**) the *y* direction, and (**f**) total magnitude after correcting measurements of aperture positions from an array that we pattern by decreasing the electron-beam current from 1.0 nA to 0.125 nA, maintaining the dwell time, and taking eight passes to deliver the same dose

Our new measurement capability closes the gap between common optical microscopes and uncommon instruments for dimensional metrology^53^, and is immediately applicable to new tests of aperture arrays. For example, using widefield measurements, we can rapidly quantify the dependence of placement precision on fabrication parameters such as dose rate. We decrease the electron-beam current and increase the dwell time by a factor of five with respect to the standard process. The standard deviation of position errors in the *x* direction increases, as Table 1 and Fig. 8a–c show, indicating an asymmetry of our lithography system and that placement precision degrades with decreasing dose rate. Second, we reduce the dwell time by a factor of eight, and overwrite the pattern eight times. The standard deviation of position errors decreases in the *y* direction, but systematic effects increase this value in the *x* direction, as Table 1 shows, and a striation pattern emerges, as Fig. 8d–f shows. This pattern further indicates an asymmetry of our lithography system and that aperture placement errors compound with pattern overwriting. Interestingly, regions of Fig. 8d, f show systematically smaller position errors, indicating a useful anomaly of the patterning process. These results are all roughly consistent with the specification of beam positioning of 2 nm for our lithography system, but manifest unpredictable irregularities. The high speed and low cost of critical-dimension localization microscopy would facilitate quality control of aperture arrays in their production as reference materials.

### Nanoparticle fiducials

Transillumination of the aperture array produces an array of point sources that are static with respect to the imaging substrate at any scale that is relevant to our measurements, providing a stable reference material for evaluating any apparent motion of fluorescent nanoparticles as fiducial markers. We localize apertures or nanoparticles in an image series, and assess the apparent motion of each point source using two-dimensional rigid transformations to register corresponding points in image pairs. We quantify apparent motion as the standard deviation of the registration errors over $\sqrt 2$. Further details are in Supplementary Note S10. This analysis eliminates unintentional motion of the measurement system in the *x* and *y* directions, but not in the *z* direction, as a source of error. For static point sources of one color, registration errors are due only to empirical localization precision and random components of localization error. Normalization of this value by theoretical localization precision allows for direct comparison of nanoparticles and apertures. The aperture array then allows for assessment of additional apparent motion. Any such motion of nanoparticles that exceeds that of apertures is due to actual motion. In this evaluation, the time that is necessary for our microscope to image through focus provides an experimental boundary between faster and slower time scales.

Rigid registration of consecutive images enables tests of motion at a time scale of 10^−1^ s. Apertures show apparent motion that ranges from 0.30 nm to 0.65 nm in a single lateral dimension, or a factor of 1.2 to 2.0 times the Cramér–Rao lower bound for each aperture, as Supplementary Fig. S19 shows. For fluorescent nanoparticles on a microscope coverslip, apparent motion ranges from 0.30 nm to 0.85 nm, or a factor of 1.2 to 1.9 times the Cramér–Rao lower bound for each nanoparticle, as Supplementary Fig. S19 shows. These values exceed the Cramér–Rao lower bound by amounts that are consistent with random components of localization error, demonstrating that the nanoparticles do not move in any way that we can measure at this time scale.

**Fig. 9:**
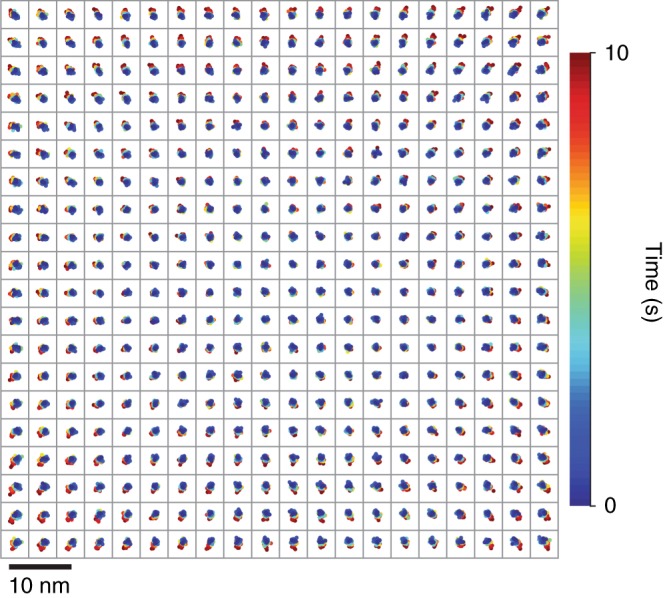
Apparent motion. Grid of scatterplots, each corresponding to a single aperture, showing apparent radial motion due to unintentional motion of the measurement system in the *z* direction over 10^1^ s. The grid spacing indicates an aperture array pitch of 10 µm. The scale bar of 10 nm corresponds to the scatterplots

Rigid registration of each image in a time series with respect to the first image extends the time scale to 10^1^ s. At this time scale, apertures appear to move radially, with registration errors that increase with distance from the center of the field, as Figure 9 shows. Imaging through focus results in apparent motion^54^ that is qualitatively similar, as Supplementary Fig. S20 shows, indicating that this apparent motion is consistent with unintentional motion of the measurement system in the *z* direction.

At slower time scales, imaging through focus decreases unintentional motion in the *z* direction to less than 10 nm. Selection of the *z* position that minimizes registration error, as Supplementary Fig. S8 shows, complements other active^39^ and passive^47^ methods for mitigating instability of *z* position. Over 10^4^ s, both apertures and nanoparticles exhibit apparent motion that is quantitatively consistent within their respective mean values of empirical localization precision of approximately 0.43 nm for apertures and 0.55 nm for nanoparticles, as Supplementary Fig. S21 shows. This apparent motion is likely due to differences in *z* position that are below the positioning resolution between images. Considering that the apertures are static, we conclude that the nanoparticles are static.

These results introduce a new capability for answering open questions about the apparent motion of fluorescent nanoparticles relative to imaging substrates. For an experimental system that is representative of common practice, in that it makes use of typical materials and methods and nonspecific binding, we find that fluorescent nanoparticles can function as fiducial markers with subnanometer stability for several hours. Previous studies reporting nanoparticle motion have not fully characterized the interactions of the components of the measurement system, in particular, unintentional motion along the optical axis, using a stable reference material such as an aperture array. It is evident from our study that this source of motion of any fiducial is clearest across a wide field and upon comparison with other fiducials in an array and is less apparent across a smaller field or at the field center.

## Conclusions

It is remarkable that the optical microscope, which has for centuries enabled observations at the micrometer scale, can potentially enable localization measurements at the atomic scale across a millimeter field. In such measurements, localization precision is largely a function of emitter intensity and stability, but localization accuracy depends on a comprehensive calibration of the parts of a measurement system and their interaction. Such calibration is rarely, if ever, implemented, which can cause gross overconfidence in measurement results with small statistical uncertainties but large systematic errors that vary across the imaging field. Such false precision is becoming increasingly problematic as measurements achieve empirical localization precision at the nanometer scale, imaging fields extend into the millimeter scale, and multifocal^55^ and multicolor^56^ methods emerge to exploit such fields. In this article, we have revealed the surprising extent of this widespread problem and presented a practical solution to it, advancing the practice of localization microscopy.

We have developed the aperture array into a multifunctional reference material that is usefully accurate, precise, planar, and stable. By a combination of widefield and scanning measurements, we have calibrated our microscope system and characterized our aperture arrays. For the first time, we have demonstrated subnanometer localization error across a submillimeter field, for multiple colors and emission sources. This new capability has enabled two novel applications. First, critical-dimension localization microscopy facilitates rapid characterization of aperture arrays by widefield imaging, allowing for the study of nanofabrication processes and quality control of reference materials for microscope calibration. Second, we exploit the stability of aperture arrays to evaluate the stability of nanoparticle fiducials, which multiple studies have called into question. We find that microscope instability can obscure the true stability of fluorescent nanoparticles on an imaging substrate, and we provide a method for evaluating different systems.

Our study motivates future work including characterization of aperture arrays by other forms of critical dimension metrology, integration of aperture arrays with various sample environments, and fabrication of other types of reference materials for localization microscopy.

## Electronic supplementary material

Supplementary information
