# Supplementary material for: Subnanometer localization accuracy in widefield optical microscopy
Source: Light Sci Appl. 2018 Jul 11;7:31. doi: 10.1038/s41377-018-0031-z (PMC6107003; doi:10.1038/s41377-018-0031-z)
Supplement: Supplementary file 1 — Supplementary information [file 41377_2018_31_MOESM1_ESM.pdf]

Supplementary information *for* Subnanometer localization accuracy in widefield optical microscopy

Craig R. Copeland[1, 2], Jon Geist[3], Craig D. McGray[3], Vladimir A. Aksyuk[1], J. Alexander Liddle[1], B. Robert Ilic[1], and Samuel M. Stavis[1, *]

[1]Center for Nanoscale Science and Technology, National Institute of Standards and Technology, Gaithersburg, Maryland 20899, [2]Maryland NanoCenter, University of Maryland, College Park, Maryland 20742, [3]Engineering Physics Division, National Institute of Standards and Technology, Gaithersburg, Maryland 20899, [*]sstavis@nist.gov

INDEX

Note S1. Aperture array – fabrication
Table S1. Aperture array – characteristics
Note S2. Aperture array – characterization
Fig. S1. Aperture array – electron microscopy
Fig. S2. Aperture array – interferometric optical microscopy
Fig. S3. Aperture array – optical microscopy
Fig. S4. Aperture array – point spread functions
Fig. S5. Nanoparticle fiducials
Fig. S6. LED and dye spectra
Note S3. Sample leveling
Fig. S7. Sample leveling
Note S4. Optimal focus
Fig. S8. Optimal focus
Fig. S9. Dark calibration of camera
Table S2. Terminology
Fig. S10. Light calibration of camera
Note S5. CMOS localization
Table S3. CMOS localization
Note S6. Localization algorithms
Fig. S11. Localization algorithm performance
Table S4. Localization algorithm performance
Note S7. Point source test
Fig. S12. Objective lenses
Table S5. Objective lenses
Fig. S13. Error correction depends on z position
Fig. S14. Error correction across the aperture array
Note S8. Scanning and widefield measurements
Table S6. Pitch variability
Table S7. From pitch variance to position standard deviation
Table S8. Effects of chromatic aberration
Fig. S15. Registration errors from three colors at one focal plane
Fig. S16. Registration errors from two colors at optimal focal planes
Note S9. Error analysis for multicolor registration
Table S9. Empirical localization precision in multicolor registration
Table S10. Localization error in multicolor registration
Fig. S17. Correction of fluorescence data
Fig. S18. Pitch across the aperture array
Table S11. Pitch characterization for two lithography systems
Note S10. Rigidity analysis
Fig. S19. Nanoparticle stability down to 10^{-1} s
Fig. S20. Apparent motion
Fig. S21. Nanoparticle stability up to 10^{4} s

Note S1. Aperture array – fabrication

We begin with silica substrates with manufacturer specifications of thickness of approximately 170 µm, surface roughness of less than 0.7 nm root mean square, scratch number of 20, dig number of 10, flatness deviation from 2.5×10^{-4} nm·nm^{-1} to 5.0×10^{-4} nm·nm^{-1}, and a parallelism of better than 0.15 mrad. We deposit a titanium film with a thickness of approximately 10 nm as an adhesion layer, a platinum film with a thickness of approximately 80 nm for optical opacity, a positive-tone electron-beam resist film with a thickness of approximately 120 nm, and an aluminum film with a thickness of approximately 15 nm for charge dissipation.

We use two electron-beam lithography systems, enabling comparison of independent aperture arrays to test placement accuracy, and fabrication of different types of aperture arrays that use and test the different operating modes of the systems. Other than different load locks, the lithography systems have nearly identical hardware. Each system has a scanning stage with two laser interferometers to measure stage position in the x and y directions. The resolution of a stage position measurement is 632.8 nm / 1024 = 0.6180 nm, with traceability to the SI through the operating wavelength of the helium–neon laser. One lithography system operates four of five electron-optical lenses and has a write field of 1 mm by 1 mm, which is useful to avoid stitching errors in patterning aperture arrays for widefield imaging, and has a specification for beam placement of 2 nm. The electron-beam current for this system is typically 1.0 nA, although we reduce it in some tests of patterning parameters that we note. The other lithography system operates five of five electron-optical lenses and has a better specification for beam placement of 0.125 nm, which nominally improves placement precision, but does so over a smaller write field of 62.5 µm by 62.5µm. The electron-beam current for this system is 1 nA. We perform a Monte Carlo simulation of electron trajectories in the film stack to correct the pattern data for proximity effects at an accelerating voltage of 100 kV, and we fracture the pattern data into polygons.

After electron-beam exposure, we remove the aluminum film with tetramethylammonium hydroxide and cold-develop the electron-beam resist in hexyl acetate. Finally, we mill the apertures with argon ions, using a secondary-ion mass spectrometer to monitor emission products and stop at the top surface of the silica substrate. The electron-beam resist is not trivial to remove after argon-ion milling and does not affect the function of the aperture array in any way that we are aware of, so we leave the resist in place.

Further characteristics of aperture arrays are in Table S1.

Table S1. Aperture array – characteristics

**Array pitch (µm)**	**Array extent (µm)**	**Nominal aperture diameter (nm)**	**Point spread function width* (pixels)**
5	350 by 350	200	1.28 ± 0.03
5	350 by 350	300	1.24 ± 0.02
5, 10	350 by 350	400	1.27 ± 0.02
5	350 by 350	500	1.37 ± 0.01
5	62.5 by 62.5	500	1.39 ± 0.01

*We characterize the width of the point spread function as $(\sigma_x + \sigma_y)/2$, as Note S7 describes in more detail. Uncertainties are one standard deviation. The mean size of image pixels is approximately 100 nm.

Note S2. Aperture array – characterization

We inspect the standard aperture array by scanning electron microscopy, as Fig. S1 shows, at an accelerating voltage of 1 kV and using an Everhart-Thornley detector at a working distance of 9 mm. The apertures are approximately circular with shape irregularity at the scale of tens of nanometers and nonvertical sidewalls, resulting in functional diameters at the silica surface that are apparently smaller than the nominal diameters. We do not attempt to quantify array pitch from these electron micrographs. To do so at the relevant scale would require calibration of the electron microscope and localization analysis that are beyond the scope of this study.

We measure the upper surface topography of the standard aperture array by interferometric optical microscopy, as Fig. S2 shows, at a peak wavelength of 475 nm with a bandwidth of 125 nm. The z position of the piezoelectric stage of this microscope is traceable to the SI through a reference material for step height, and we further calibrate these measurements using a reference flat of silicon carbide. We extract the center of the interference pattern as a function of z position as the location of the reflecting surface. We fit the resulting upper surface topography of the aperture array to a plane to level it and analyze the z-position variation of the upper surface as an indicator of the lower interface between silica and titanium. We expect and observe scratches and digs consistent with the polish of the silica substrate transferring through conformal films. The standard deviation of z position is 1.76 nm, such that the upper surface is effectively flat within the z-position resolution of 10 nm of our localization microscope. Therefore, in subsequent analysis, we ignore any nonflatness of the aperture array. However, in the production of reference materials for localization microscopy in three dimensions, this issue motivates the use of even flatter substrates, or the characterization and analytical correction of any nonplanar surface topography of the aperture array.

After developing our localization measurements and analyses, we apply them to test the extent to which apertures of varying nominal diameters appear as point sources. We summarize these results in Table S1, and describe them in more detail in Note S7. These results indicate that the apertures have functional diameters that are smaller than their nominal diameters, or that our microscope system does not achieve its expected spatial resolution, or a combination of these two effects.

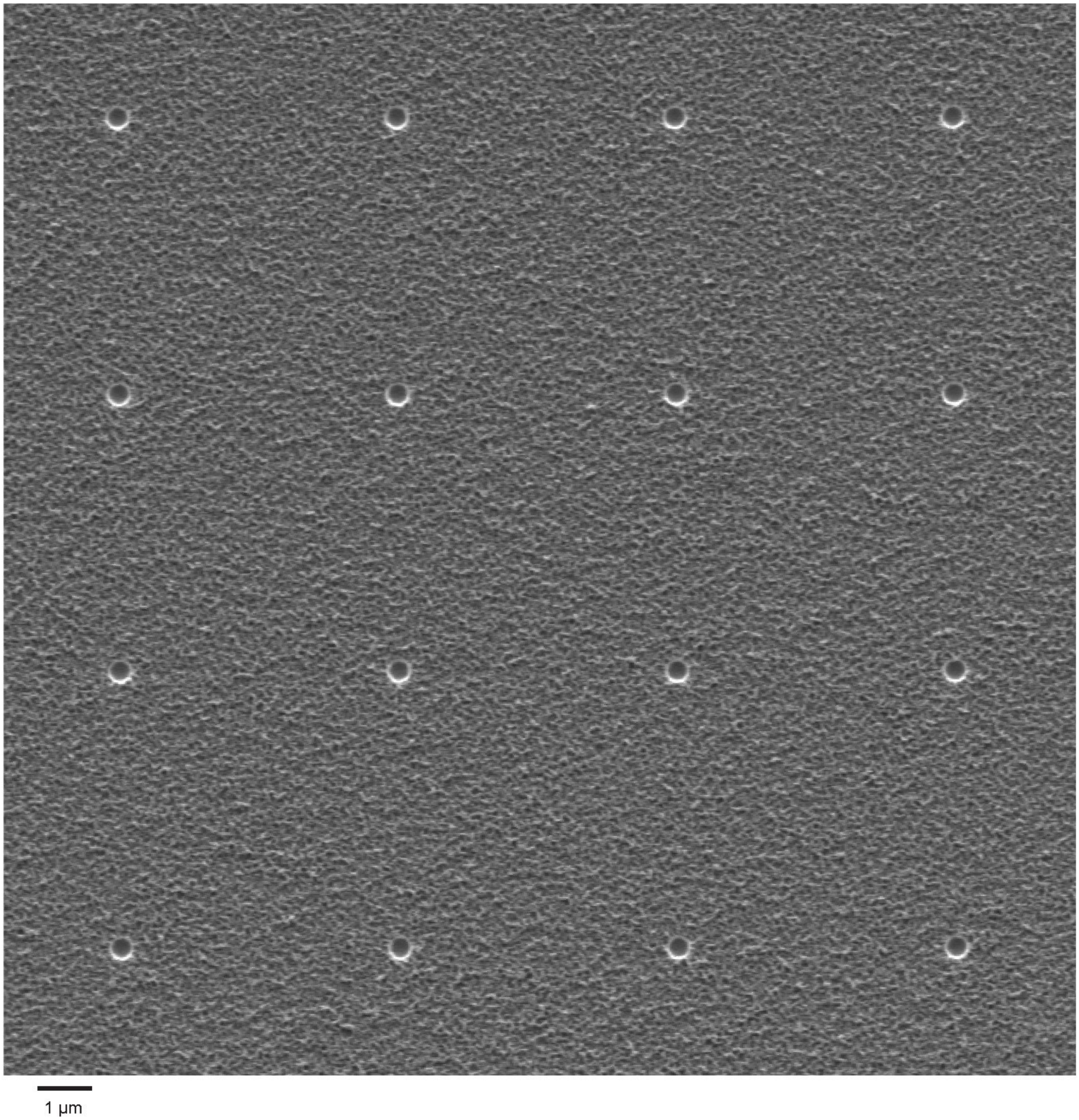

Figure S1. Aperture array – electron microscopy. Scanning electron micrograph showing 16 apertures. Surface texture around the apertures is from electron-beam resist.

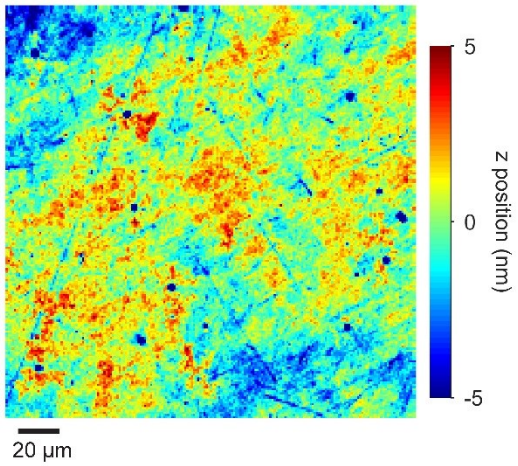

Figure S2. Aperture array – interferometric optical microscopy. Interferometric optical micrograph showing the upper surface topography of a representative region corresponding approximately to the aperture array. The apertures are below the resolution of this imaging system. Scratches and digs in the upper surface are consistent with the polish of the lower silica surface. The standard deviation of z position is 1.76 nm.

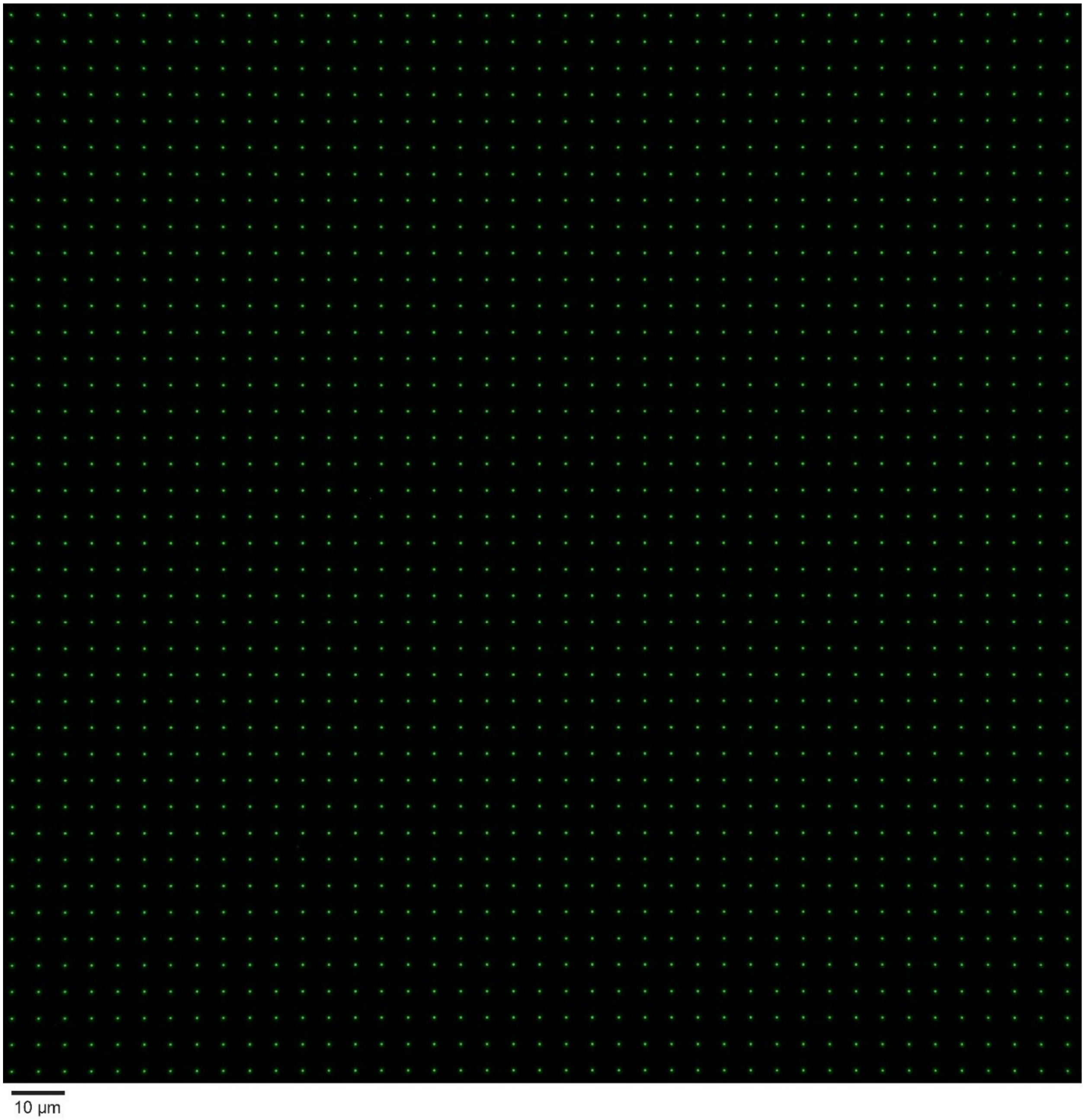

Figure S3. Aperture array – optical microscopy. Brightfield optical micrograph showing the transmission of light through an aperture array over the full field of the imaging system of approximately 200 µm by 200 µm. False color represents the illumination wavelengths of around 500 nm.

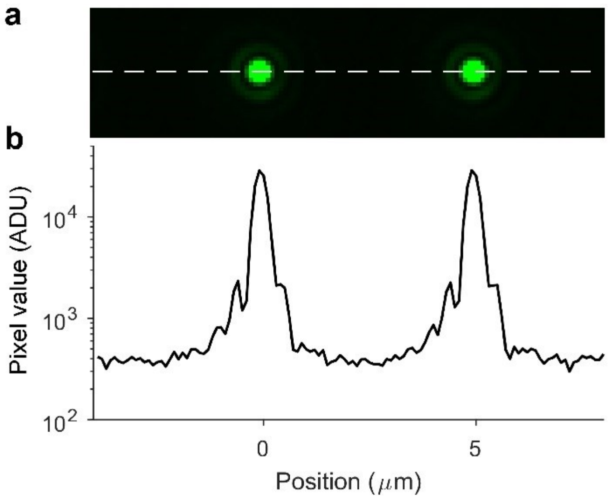

Figure S4. Aperture array – point spread functions. **(a)** Brightfield optical micrograph showing the point spread functions from two apertures with nominal diameters of 400 nm in an array with a nominal pitch of 5 µm. **(b)** Plot showing pixel value along the white dashed line in (a). Airy rings are evident on a logarithmic scale for the vertical axis. The point spread function from the left aperture decays to background by approximately 3 µm from the center position of the aperture. This shows that an array pitch of 5 µm provides sufficient separation of adjacent apertures such that their signals do not appreciably overlap within the region of interest for localization analysis, which is approximately 1 µm around the center position of each aperture.

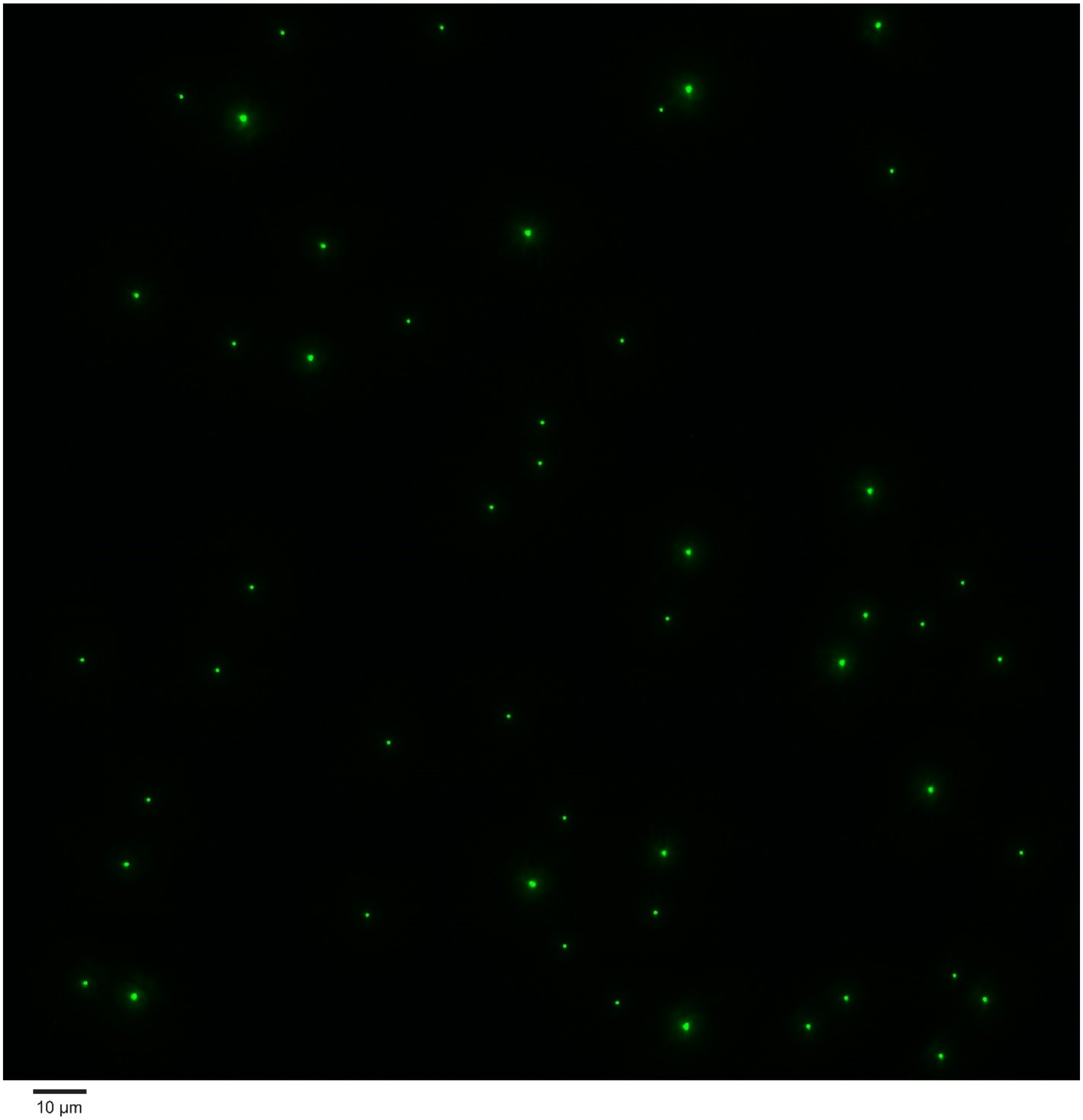

Figure S5. Nanoparticle fiducials. Fluorescence micrograph showing fluorescent nanoparticles with a carboxylate coating on a borosilicate coverslip with a poly-D-lysine coating. In subsequent analysis, we ignore aggregates of nanoparticles, which are evident as images that are brighter and larger than single point spread functions.

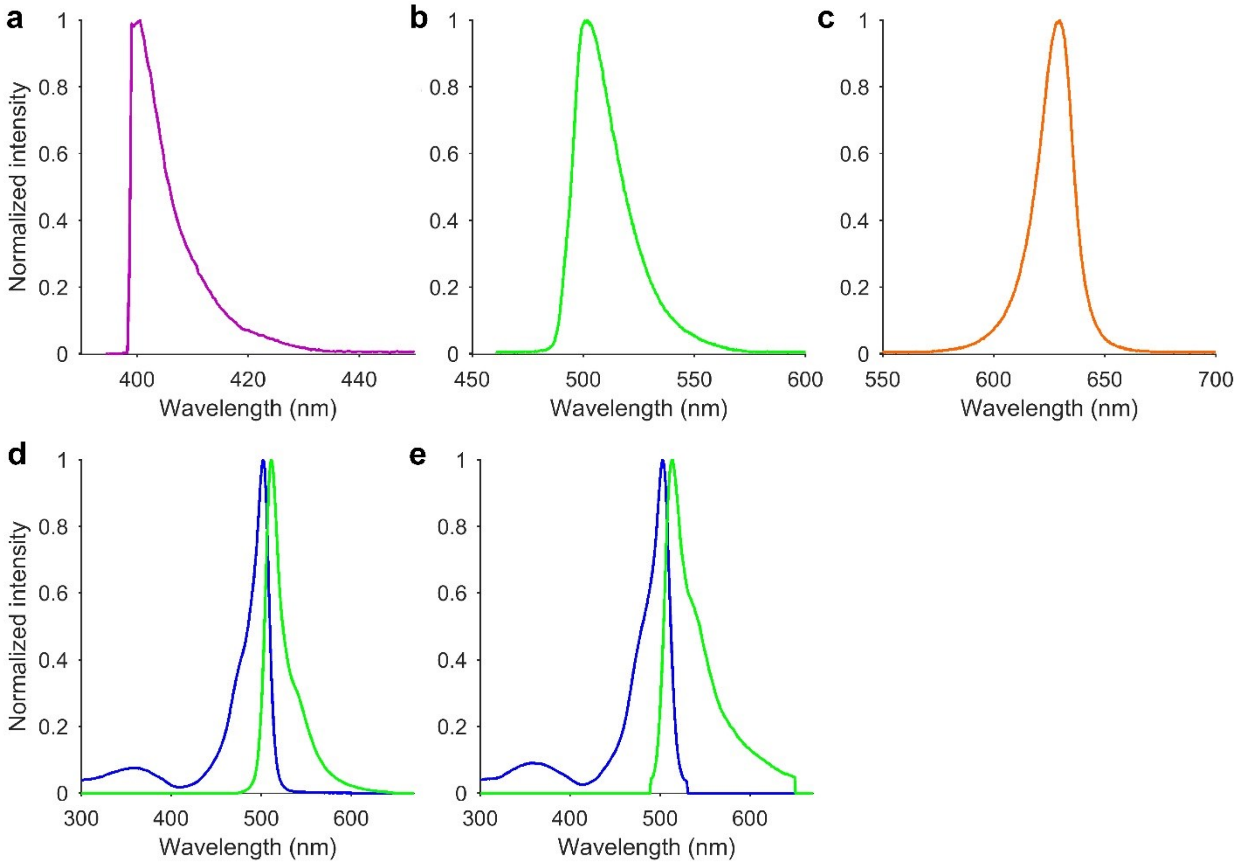

Figure S6. LED and dye spectra. (**a**-**c**) Plots showing experimental emission spectra of LED arrays with peak wavelengths of (a) 400 nm, (b) 500 nm, and (c) 630 nm. (**d-e**) Plots showing nominal excitation (blue) and experimental emission (green) spectra of (d) boron-dipyrromethene dye in N,N-dimethylformamide solution and (e) in amorphous polystyrene nanoparticles.

Note S3. Sample leveling

We can level a sample by aligning its surface normal to the optical axis using two methods. The first exploits piezoelectric actuation and characterization of the z position of the objective lens, as we describe in the main text. The second takes advantage of Zernike theory. Both require a stage insert that enables rotation of the sample about the x and y axes, as Fig. S7a-b shows. In the second method, we analyze spatial maps of ρ, as we define in Eq. 1 and Eq. S1, across the field. We fit the maps to a linear combination of Zernike polynomials[1] in real time, finding the optimal orientation which minimizes the coefficients for the first-order Zernike polynomials Z_1^1 and Z_1^{-1}, which model orientation of the sample about the x and y axes, as Fig. S7b-f show.

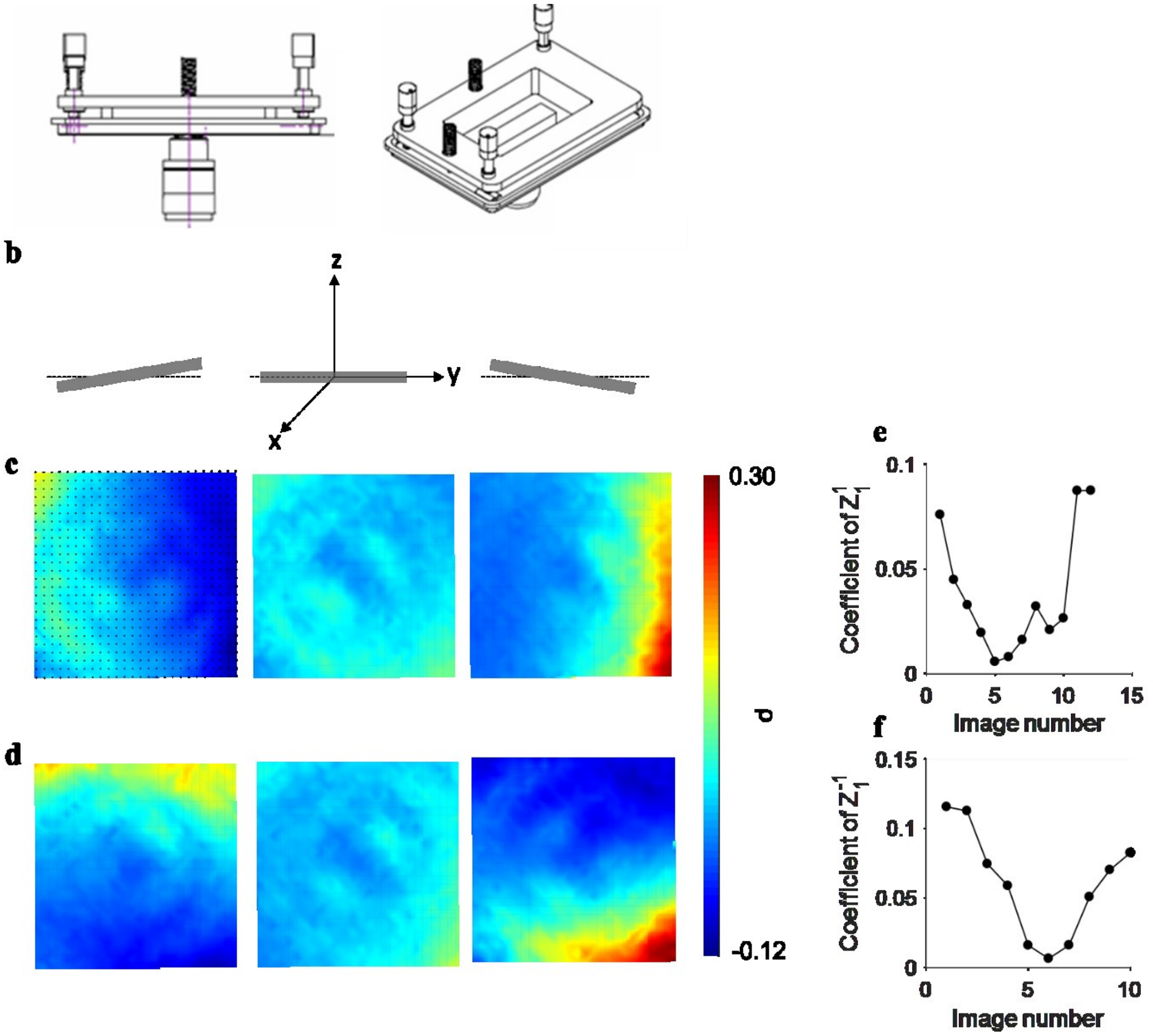

Figure S7. Sample leveling. (**a**) Schematic showing sample holder. **(b)** Schematic showing sample orientation about the x axis, not to scale. **(c)** Plots showing ρ at varying magnitudes of orientation about the x axis. Black dots indicate aperture positions. **(d)** Plots showing ρ at varying magnitudes of orientation about the y axis. Orientation direction corresponds to the schematics in (b). **(e)** Plot showing representative values of the coefficient of the Zernike polynomial Z_1^1, modeling orientation about the x axis. The minimum corresponds to the center plot in (c). **(f)** Plot showing representative values of the coefficient of Z_1^{-1}, modeling orientation about the y axis. The minimum corresponds to the center plot in (d).

Note S4. Optimal focus

For any region of interest, from a square micrometer around a single aperture to the full field of the imaging system, we determine optimal focus first by imaging through focus. We then extract the mean amplitude of the point spread functions that are within the region of interest and empirically model the variation of the mean amplitude with respect to z position using a quintic function. We take the maximum value of the model fit as the z position of optimal focus. Fig. S8 shows amplitude as a function of z position for one aperture and mean amplitude as a function of z position for many apertures in one image.

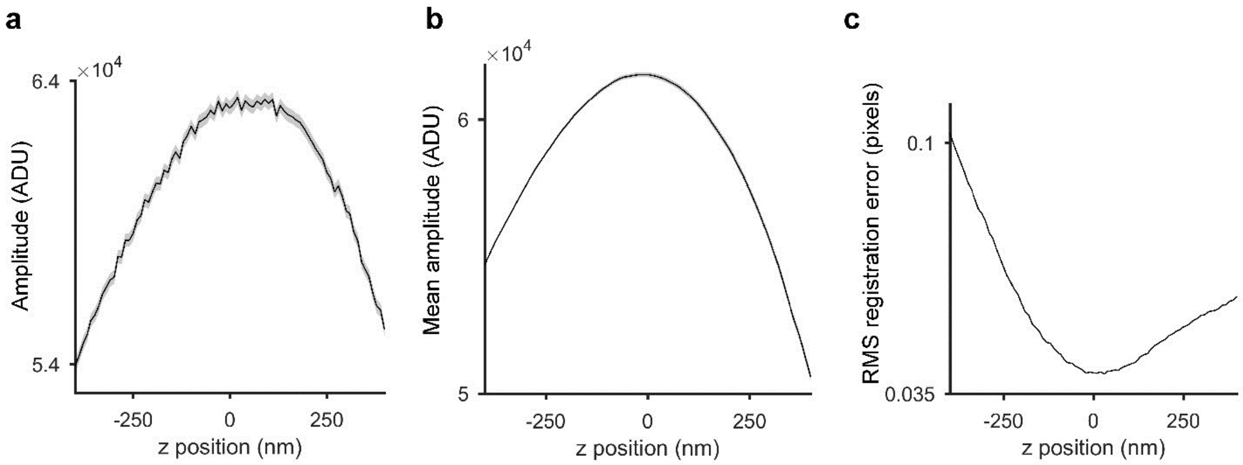

Figure S8. Optimal focus. **(a)** Plot showing the amplitude of the point spread function of a single aperture as a function of z position, with a maximum at optimal focus. The grey boundary is one standard deviation. **(b)** Plot showing the mean amplitude of 1 600 point spread functions from as many apertures as a function of z position, with a maximum at the optimal focal plane. The z position of optimal focus of the aperture in (a) differs from the z position of the optimal focal plane in (b) due to field curvature. **(c)** Plot showing the root-mean-square error of a rigid registration between images of an aperture array as a function of z position, with a minimum at the z position of the common optimal focal plane between the two images. The grey boundaries in (b) and (c) are one standard error and are comparable in width to the black lines.

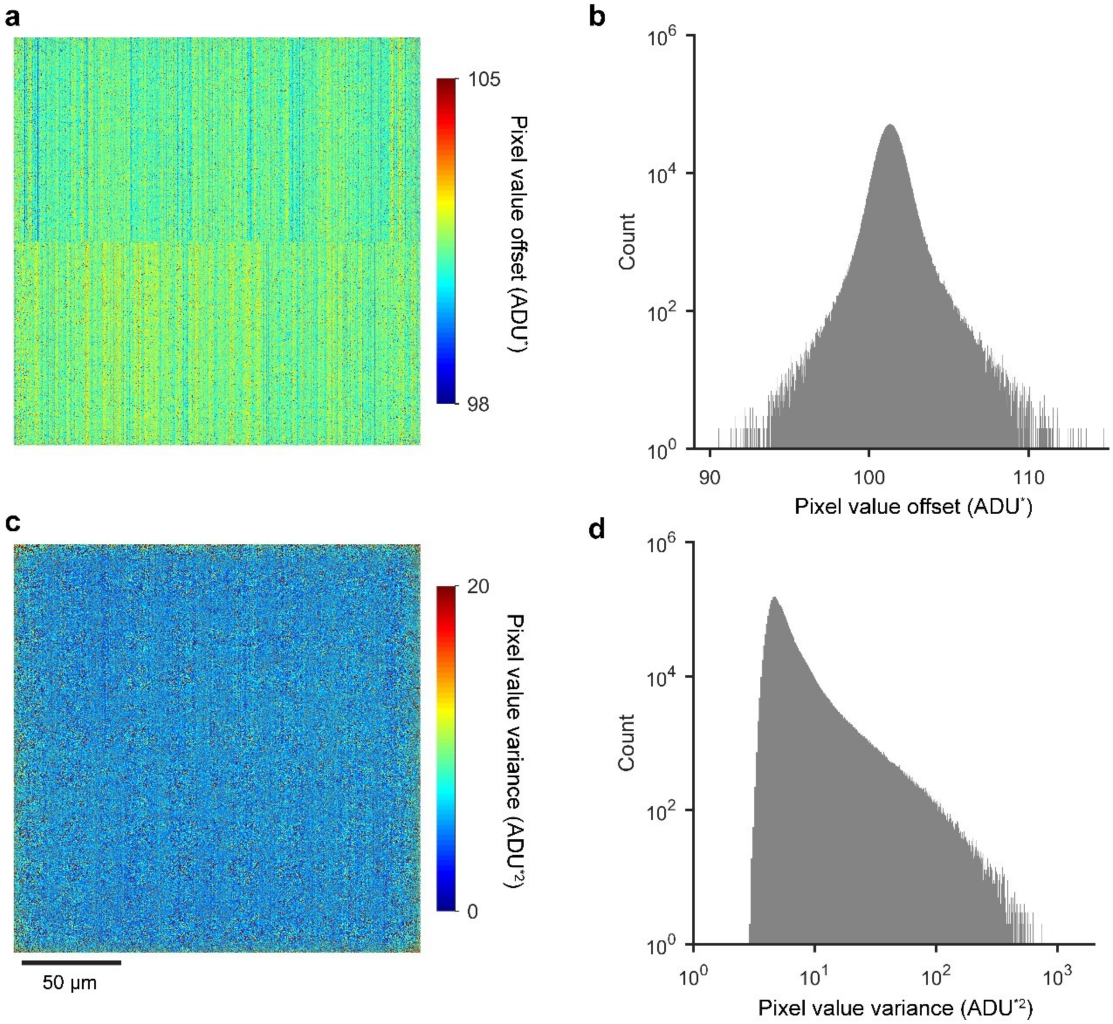

Figure S9. Dark calibration of camera. Initially, pixel values are in analog-to-digital units before calibration (ADU*). (**a**) Plot showing pixel value offset. (**b**) Histogram showing pixel value offset. (**c**) Plot showing pixel value variance. (**d**) Histogram showing pixel value variance. To clearly show systematic effects in (a) and (c) from the CMOS architecture of the imaging sensor, we restrict the ranges of (a) with respect to (b) and (c) with respect to (d).

Table S2. Terminology

Process	Term	Sources of Error	Quantity
Aperture fabrication	Placement accuracy	Electron-optical aberrations<br>Position resolution of lithography system	Mean magnitude of differences of aperture placements from nominal positions*
	Placement precision	Pattern resolution and transfer	Standard deviation of difference of aperture placements from nominal positions*
Emitter localization	Theoretical localization precision	Photon shot noise<br>Background noise<br>Image pixel size<br>Point spread function	Cramér–Rao lower bound
	Empirical localization precision	Theoretical localization precision<br>Fitting error<br>Unintentional random motion of measurement system	Standard deviation of difference of position measurements from mean value of position measurements
Microscope calibration	Position accuracy	Placement precision<br>Photon-optical aberrations<br>Image pixel size<br>Fitting error<br>Unintentional systematic motion of measurement system<br>Empirical localization precision	Position error – difference of aperture position measurement from nominal position*
	Correction accuracy	Placement precision	Correction error – difference of placement precision and the standard deviation of position errors in a synthetic array with ideal placement accuracy
Error correction	Localization accuracy	Unintentional axial motion of measurement system<br>Correction accuracy<br>Unknown sources of error	Localization error – standard deviation of position errors, independent of placement precision and empirical localization precision
Data registration	Registration accuracy	All sources above<br>Chromatic aberration	Registration error – difference of corresponding position measurements from two images

*Nominal positions are at the nodes of an ideal square array as per our design. Mean differences that do not alter the mean value of array pitch do not affect microscope calibration.

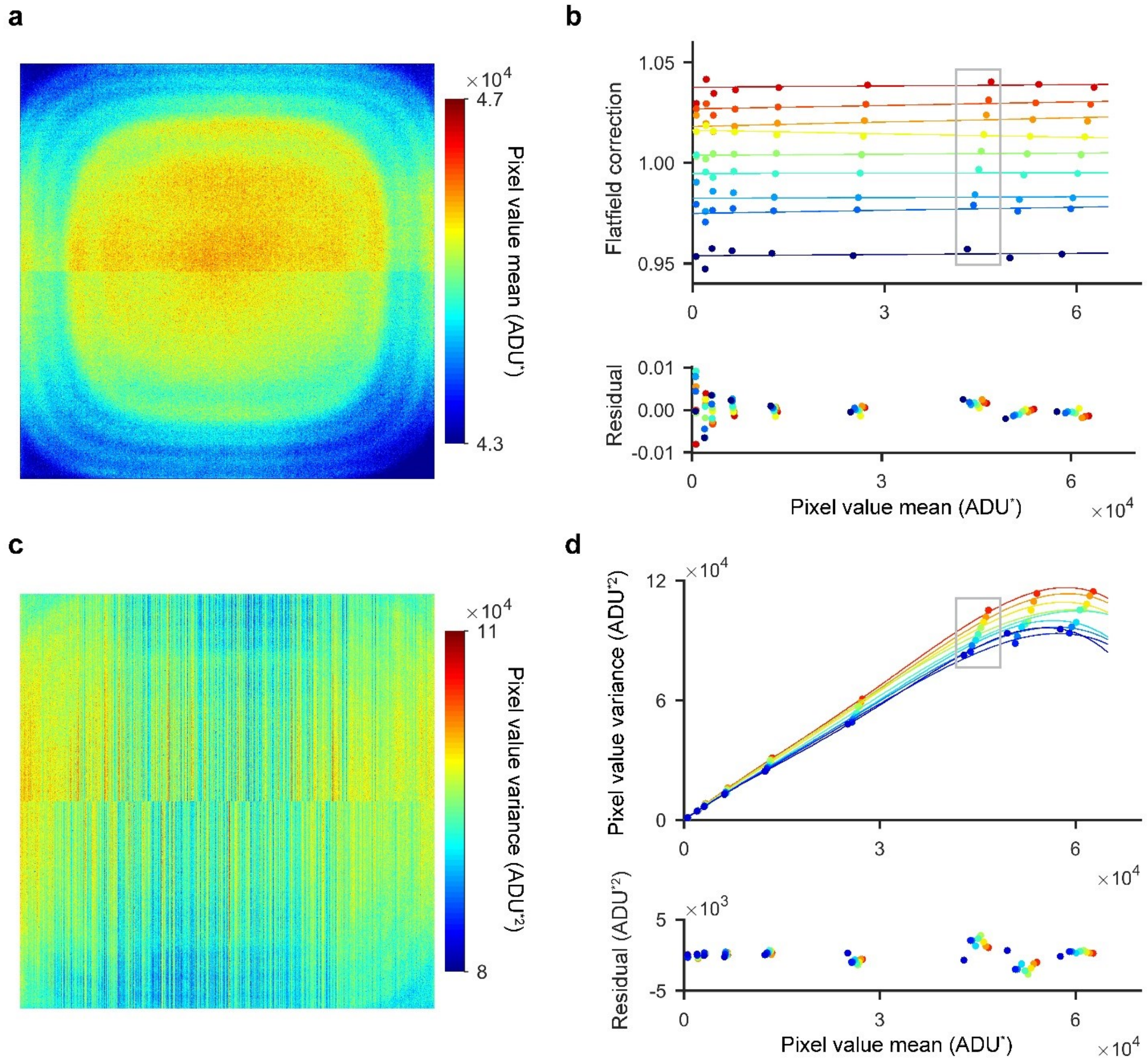

Figure S10. Light calibration of camera. (**a**) Plot showing pixel value mean from 15000 images at one of nine illumination levels. Nonuniformity results from the illumination profile, sensor packaging, and CMOS architecture. (**b**) Plot showing flatfield corrections for nine representative pixels as a function of pixel value mean. The gray box encloses data from the illumination level in (a). The flatfield corrections abruptly increase at low values and then remain nearly constant for the remaining 95% of the dynamic range. A linear function empirically approximates the flatfield corrections over the full dynamic range. (**c**) Plot showing pixel value variance corresponding to the pixel value mean in (a). Nonuniformity results from sensor packaging and amplifier columns. (**d**) Plot showing pixel value variance, including contributions from shot noise, read noise, and fixed-pattern noise, as a function of pixel value mean for nine representative pixels. The gray box encloses data from the illumination level in (a, c). A quartic polynomial empirically approximates the pixel value variance over the full dynamic range. The ratio of pixel value variance to pixel value mean gives an approximate value of gain. Therefore, the quartic polynomial can provide an estimate of gain for any pixel and pixel value, without flatfield correction, to convert units from ADU* to photons, such as for calculation of a Cramér–Rao lower bound.

Note S5. CMOS localization

We test localization accuracy for single emitters over the full dynamic range and field of our CMOS camera. We model the response of each pixel as a Gaussian probability density function, which replaces the Poisson distribution that commonly models shot noise,[2, 3, 4] due to the nonlinear relationship between pixel value and total variance. The probability density function for each pixel incorporates the pixel value offset and flatfield correction in the calculation of the mean or expected pixel value to account for variation in pixel gain, illumination nonuniformity, and the effects of sensor packaging. The variance of the probability density function comes from the quartic function in the main text. We perform Monte Carlo simulations to generate images of a univariate Gaussian point spread function in which this same Gaussian probability density function, incorporating parameter values that correspond exactly to a region of our CMOS camera, determines each pixel value. This analysis results in accurate localization with uncertainties near the Cramér–Rao lower bound, as Table S3 shows for the x direction. We find that using an approximate model for total variance, which includes only contributions from shot noise and read noise for each pixel, results in empirical localization precision and localization accuracy that are equivalent to using the empirical model for the total variance. This demonstrates that, despite the difference between the empirical and approximate variance, which is significant for pixels with values in the top 25% of the dynamic range, the approximate model is more efficient and is equally accurate even for images of point sources with pixel values that span the full dynamic range of the CMOS sensor.

Table S3. CMOS localization

**Number of signal photons**	**Theoretical localization precision (pixels)**	**Empirical localization precision* (pixels)**	**Standard error* (pixels)**	**Empirical error* (pixels)**
4.5×10^{5}	2.7×10^{-3}	2.9×10^{-3}	4.1×10^{-5}	5.8×10^{-5}
7.0×10^{5}	2.2×10^{-3}	2.4×10^{-3}	3.4×10^{-5}	5.3×10^{-5}

*Values from measurements of 5000 images.

Note S6. Localization algorithms

We approximate the point spread function, which varies across the imaging field, with a bivariate Gaussian function,

$$G_{\mathrm{biv}}\big(x, y, \Theta = [\mathrm{A}, \sigma_x, \sigma_y, \rho, x_0, y_0, C]\big) = A \cdot \exp - \left(\frac{1}{2(1-\rho^2)}\left[\frac{(x-x_0)^2}{\sigma_x^2} - 2\rho\frac{(x-x_0)(y-y_0)}{\sigma_x\sigma_y} + \frac{(y-y_0)^2}{\sigma_y^2}\right]\right) + C, \quad \text{(Eq. S1)}$$

where A is the amplitude, x_0 is the position of the peak in the x direction, y_0 is the position of the peak in the y direction, σ_x is the standard deviation in the x direction, σ_y is the standard deviation in the y direction, ρ is the correlation coefficient between the x and y directions, and C is a constant background. This model determines the expected pixel value in analog-to-digital units (ADU) for each pixel in an image,

$$E_i(x_i, y_i, \Theta) = G_{\mathrm{biv}}(x_i, y_i, \Theta), \quad \text{(Eq. S2)}$$

where i indexes each pixel, x_i is the position of the pixel in the x direction, y_i is the position of the pixel in the y direction. For weighted least-squares, the objective function for fitting this model of the expected pixel values using is,

$$\widehat{\Theta} = \operatorname{argmin}\left[\sum_i \frac{(I_i - E_i)^2}{gI_i + \sigma_{\mathrm{read},i}^2}\right], \quad \text{(Eq. S3)}$$

where $\widehat{\Theta}$ is the estimate for the parameter set $\widehat{\Theta} = \{A, \sigma_x, \sigma_y, \rho, x_0, y_0, C\}$,

g is the nominal gain of the camera specified by the manufacturer, $\sigma^2_{\mathrm{read},i}$ is the pixel read noise, and I_i is the experimental pixel value after calibration for CMOS characteristics,

$I_i = \frac{I_i^* - o_i}{FF_i}$, (Eq.S4)

where I_i^* is the pixel value before calibration, o_i is the pixel value offset, and FF_i is the flatfield correction. Subsequently, pixel values are in analog-to-digital units after calibration (ADU). In the case of a Gaussian probability density function for the response of single pixels, the objective function for maximum-likelihood is similar,

$\widehat{\Theta} = \mathrm{argmin}\left[\sum_i \frac{(I_i - E_i)^2}{gE_i + \sigma^2_{\mathrm{read},i}}\right]$, (Eq. S5)

with the only difference being the replacement of the experimental pixel value I_i in the denominator of Eq. S3 with the model or expected pixel value E_i.

If the model systematically underestimates the experimental pixel values, then the presence of the expected pixel value E_i in the denominator of Eq. S5 means that maximum-likelihood gives additional weight to the underestimated pixel, as Fig. 3 shows. In contrast, the presence of I_i in the denominator of Eq. S3 means that weighted least-squares does not have this bias. These effects are the opposite for the case that the model systematically overestimates the experimental values.

We modify our localization algorithm to mitigate such effects. A general solution to this problem of selecting either weighted least-squares or maximum-likelihood is a hybrid objective function, which empirically reduces the effect of model discrepancies whether the model systematically overestimates or underestimates the data,

$\widehat{\Theta} = \mathrm{argmin}\left[\sum_i \frac{(I_i - E_i)^2}{g \cdot \max(I_i, E_i) + \sigma^2_{\mathrm{read},i}}\right]$, (Eq. S6)

where $\max(I_i, E_i)$ reduces the weight of pixels with significant residuals. Therefore, we term this the light-weighting objective function.

We use unweighted least-squares to determine the starting point for localization with the other algorithms. The field dependence of position estimation with light-weighting, maximum-likelihood, and weighted and unweighted least-squares is in Fig. S11, and a quantitative comparison of empirical localization precision is in Table S4. We derive empirical localization precision from the standard deviation of 100 measurements in an image series of the pitch of each unit cell of the aperture array. The values in Table S4, which average over the x and y directions, are the root-mean-square of the pitch standard deviations over a factor of $\sqrt{2}$ from 1 640 pitches.

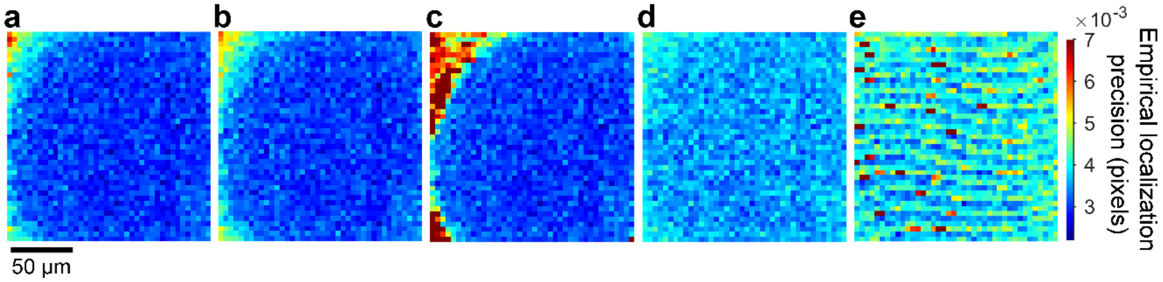

Figure S11. Localization algorithm performance. (**a-e**) Representative plots showing empirical localization precision across the field for position estimation with (a) light-weighting, (b) weighted least-squares, (c) maximum-likelihood, (d) unweighted least-squares, and (e) light-weighting with a smaller region of interest of 500 nm by 500 nm that excludes much of the point spread function outside of the central peak. The data in (e) is nearly identical for the first three localization algorithms. The mean number of signal photons per point spread function is 5.3×10^5. For this data, weighted least-squares performs similarly to light-weighting, due to deformation of the point spread function most often causing the model to underestimate the data, but this may not always be the case. Unweighted least-squares generally results in larger uncertainties than the other algorithms and is not suitable for inclusion of CMOS characteristics and shot noise. However, it is also less sensitive to the model discrepancy that Fig. 3 shows, because uniform weighting optimizes the fit to the central peak of the point spread function that is approximately Gaussian. Therefore, unweighted least-squares performs best in field regions with the largest deformation of the point spread function. Similarly, a region of interest that excludes much of the point spread function outside of the central peak results in nearly identical performance of the first three algorithms, but the empirical localization precision is significantly worse overall. The field dependence in (e) indicates systematic effects of pixelation on the definition of a localization region of interest that excludes much of the point spread function outside of the central peak. These results highlight the utility of light-weighting for accommodating deformation of the point spread function. Summary results for the different localization algorithms for different signal intensities and regions of interest are in Table S4.

Table S4. Localization algorithm performance

	**Mean number of signal photons per point spread function**			
	5.3×10^5	3.0×10^5	5.3×10^4	5.9×10^3
	**Empirical localization precision (pixels)**			
Light-weighting (Eq. S6)	0.00295 (0.00398)*	0.00399	0.00889	0.02710
Weighted least-squares (Eq. S3)	0.00301 (0.00399)*	0.00391	0.00892	0.02910
Maximum-likelihood (Eq. S5)	0.00356 (0.00399)*	0.00795	0.01398	0.03183
Unweighted least-squares	0.00339	0.00446	0.01042	0.03165

*Values in parentheses correspond to a region of interest that includes only the central peak of the point spread function.

Note S7. Point source test

We test the extent to which empty apertures with nominal diameters ranging from 200 nm to 500 nm appear as point sources under transillumination. For each value of nominal diameter, we image 400 apertures around the center of the write field and the center of the imaging field. We determine the position of optimal focus as Fig. S8 shows, localize each aperture, extract the standard deviations of the bivariate Gaussian approximation of the point spread function, and evaluate the mean value of $(\sigma_x + \sigma_y)/2$. These values are in Table S1. Apertures with nominal diameters of 200 nm, 300 nm, and 400 nm have equivalent mean values of this quantity, indicating that the functional diameters of these apertures are below the resolution of the imaging system and that they appear as point sources. These mean values of $(\sigma_x + \sigma_y)/2$ exceed the theoretical value of approximately $0.21\lambda/\mathrm{NA}$ = 90 nm, likely due to the inclusion of the first Airy ring in the fitting region of interest. Apertures with nominal diameters of 500 nm appear to be slightly larger, indicating that their functional diameters approach the resolution limit of the imaging system. On the basis of this data, in the calibration of our microscope, we typically use apertures with nominal diameters of 400 nm to maximize the number of signal photons.

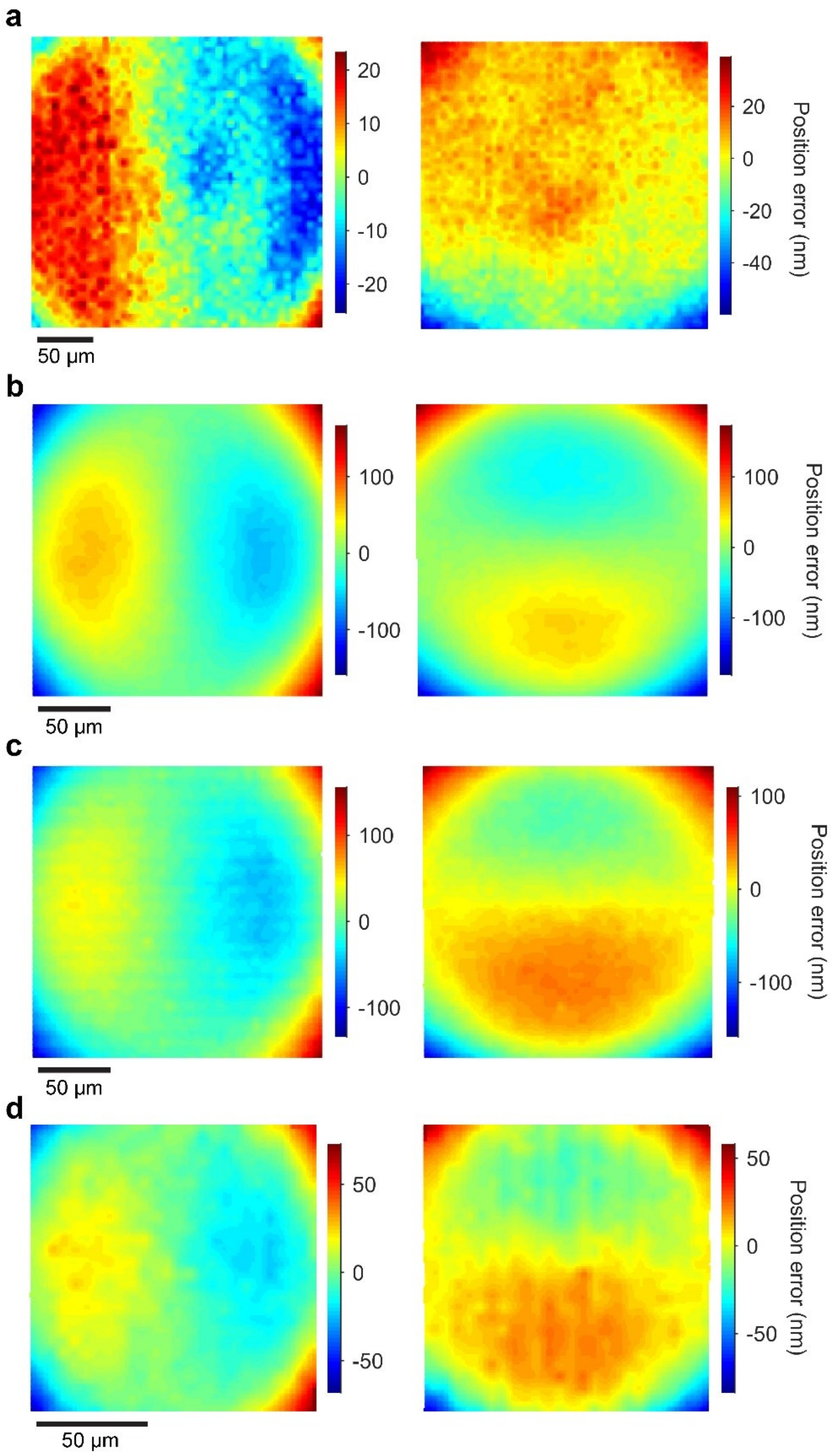

Figure S12. Objective lenses. (**a-d**) Plots showing position errors due mostly to using the mean values of image pixel size for four objective lenses with magnification and numerical aperture values of (a) 50× and 0.55, (b) 63× and 1.20, (c) 63× and 1.40, and (d) 100× and 1.46. The left column shows position errors in the x direction. The right column shows position errors in the y direction. We reconfigure the same microscope system for testing each objective lens using an

aperture array with nominal diameters of 200 nm or 400 nm. Further specifications of the objective lenses and the resulting standard deviation of position errors are in Table S5. Removing and replacing an objective lens requires recalibration of the microscope. For example, when we remove and replace the objective lens in (b), the mean value of image pixel size changes by up to 0.07%.

Table S5. Objective lenses

Magnification (×)	Numerical aperture ( )	Refractive index of immersion medium ( )	Working distance (mm)	Corrections	Standard deviation of position errors (nm)	
					x	y
50	0.55	1.00	9.1	Chromatic, flatfield	10.85 ± 0.15	11.57 ± 0.16
63	1.2	1.33	0.28	Coverslip, chromatic, flatfield	39.95 ± 0.69	39.52 ± 0.68
63	1.4	1.52	0.19	Coverslip, chromatic, flatfield	30.53 ± 0.52	30.75 ± 0.53
100	1.46	1.52	0.11	Coverslip, chromatic, flatfield	15.64 ± 0.43	16.34 ± 0.44

All objective lenses are from the same manufacturer. All specifications are nominal values from the manufacturer.

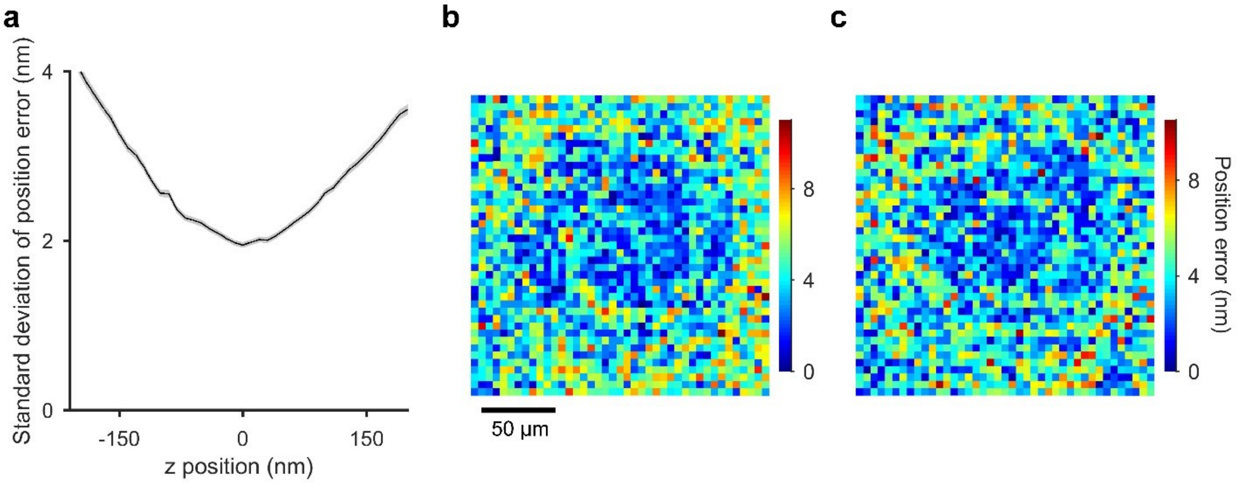

Figure S13. Error correction depends on z position. **(a)** Plot showing the pooled standard deviation of position errors in the x and y directions following error correction with respect to z position. The gray boundary is one standard error and is comparable in width to the black line. **(b, c)** Plots showing the total magnitude of position errors at (b) 150 nm below the z position of optimal focus and (c) 150 nm above the z position of optimal focus. Position errors increase with the magnitude of z position away from optimal focus, with a radial deformation of the field.

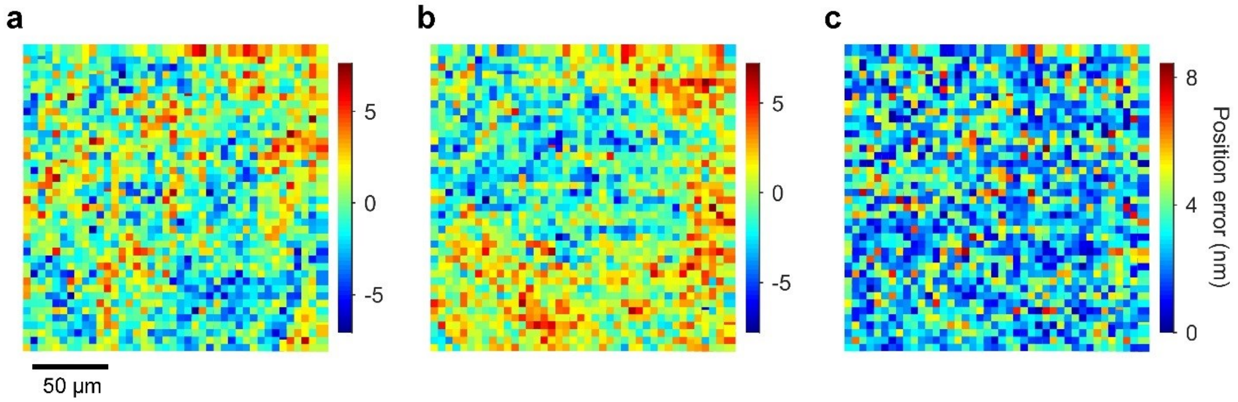

Figure S14. Error correction across the aperture array. Plots showing position errors in **(a)** the x direction, **(b)** the y direction, and **(c)** total magnitude, from applying error correction models that we derive from the center of the standard array to a different region of the standard array. Systematic effects in (b) are consistent with variation in z position with respect to the data in Fig. 5 in the main text. Additional systematic effects that may indicate the presence of electron-optical aberrations in the process of electron-beam patterning are not apparent.

Note S8. Scanning and widefield measurements

The spatial variances of pitch values across the aperture array from scanning and widefield measurements are, respectively,

$\sigma^2_{\mathrm{pitch,S}} = \sigma^2_{\mathrm{lp,S}} + \sigma^2_{\mathrm{pp}} + \sigma^2_{\mathrm{le,S}}$ (Eq. S7)

$\sigma^2_{\mathrm{pitch,W}} = \sigma^2_{\mathrm{lp,W}} + \sigma^2_{\mathrm{pp}} + \sigma^2_{\mathrm{le,W}}$ (Eq. S8)

where $\sigma^2_{\mathrm{lp,S}}$ is the variance from empirical localization precision in scanning measurements, $\sigma^2_{\mathrm{lp,W}}$ is the variance from empirical localization precision in widefield measurements, $\sigma^2_{\mathrm{le,S}}$ is the variance from localization errors in scanning measurements, $\sigma^2_{\mathrm{le,W}}$ is the variance from localization errors in widefield measurements, and σ^2_{pp} is the variance from placement precision. We determine the values of empirical localization precision from the mean variance of 1 600 pitch measurements over a time series of 100 images of the aperture array.

The difference of pitch values between scanning and widefield measurements eliminates σ^2_{pp}, isolating the independent terms in $\sigma^2_{\mathrm{pitch,S}}$ and $\sigma^2_{\mathrm{pitch,W}}$,

$\sigma^2_{\mathrm{pitch,S-W}} = \sigma^2_{\mathrm{lp,S}} + \sigma^2_{\mathrm{lp,W}} + \sigma^2_{\mathrm{le,W}} + \sigma^2_{\mathrm{le,S}}$, (Eq. S9)

and randomizing the correspondence between the scanning and widefield measurements of pitch causes σ^2_{pp} to be independent between the two measurement methods, giving a variance for the difference between the randomized pitch measurements of

$\left(\sigma^2_{\mathrm{pitch,S-W}}\right)_{\mathrm{Random}} = \sigma^2_{\mathrm{lp,S}} + \sigma^2_{\mathrm{lp,W}} + \sigma^2_{\mathrm{le,W}} + \sigma^2_{\mathrm{le,S}} + 2\sigma^2_{\mathrm{pp}}$. (Eq. S10)

Subtracting Eq. (S9) from Eq. (S10) isolates σ^2_{pp}, providing a measure of placement precision that is free from empirical localization precision and localization error. The corresponding value of placement precision is $\frac{\sigma_{\mathrm{pp}}}{\sqrt{2}}$, where dividing by $\sqrt{2}$ converts pitch standard deviation to position standard deviation. Values for these quantities are in Tables S6 and S7.

Inserting the values of σ^2_{pp} and $\sigma^2_{\mathrm{lp,W}}$ into Eq. (S8) gives a localization error in widefield measurements of $\frac{\sigma_{\mathrm{le,W}}}{\sqrt{2}}$. Values for these quantities are in Table S7. Subsequent analysis of

registration errors indicates that this calculation is conservative, as the localization error evidently includes systematic effects that cancel in registration.

Values from an analogous analysis for scanning measurements of pitch are in Table S6. The widefield values and their components in Table S6 are consistent with but slightly lower than the corresponding values in Table 1 in the main text. This is due to small differences in the characterization of position error by either the ideal array method or measurements of pitch, as well as the exclusion of shot noise.

The measurement uncertainties of variance values are the standard error of the variance as per Ref. [50] in the main text. To determine values of σ_{pp}, $\sigma_{le,W}$, and ϵ_W, we propagate uncertainty using either the NIST Uncertainty Machine, which is Ref. [52] in the main text, or the law of propagation of uncertainty.

Table S6. Pitch variability

**Measurement type**	σ^2_{pitch} **(nm^2)**	σ^2_{lp} * **(nm^2)**	σ^2_{le} **(nm^2)**
	**x direction**		
Widefield	6.83 ± 0.34**	0.184 ± 0.002**	0.78 ± 0.50***
Scanning	7.42 ± 0.37**	0.138 ± 0.0006**	1.41 ± 0.52***
	**y direction**		
I Widefield	7.73 ± 0.39**	0.154 ± 0.001**	1.03 ± 0.54***
Scanning	7.25 ± 0.36**	0.131 ± 0.0006**	0.57 ± 0.52***

*Mean variance of 800 values of pitch from a series of 100 images.
**Standard error.
***NIST Uncertainty Machine.

Table S7. From pitch variance to position standard deviation

**Quantity**	**x direction**	**y direction**
$\sigma^2_{pitch,S-W}$ (nm^2)	2.51 ± 0.13*	1.88 ± 0.09*
$\left(\sigma^2_{pitch,S-W}\right)_{Random}$ (nm^2)	14.25 ± 0.71*	14.98 ± 0.75*
σ^2_{pp} (nm^2)	5.87 ± 0.36**	6.55 ± 0.38**
$\sigma_{pp}/\sqrt{2}$ (nm)	1.71 ± 0.05**	1.81 ± 0.05**
$\sigma_{le,W}/\sqrt{2}$ (random) (nm)	0.62 ± 0.20***	0.72 ± 0.19***

*Standard error.
**NIST Uncertainty Machine.
***Propagation of uncertainty.

Table S8. Effects of chromatic aberration

**Peak wavelength (nm)**	**Mean value of image pixel size (nm)**	**Position of optimal focal plane (nm)**
400	99.85	370
500	100.01	0
630	100.13	-720

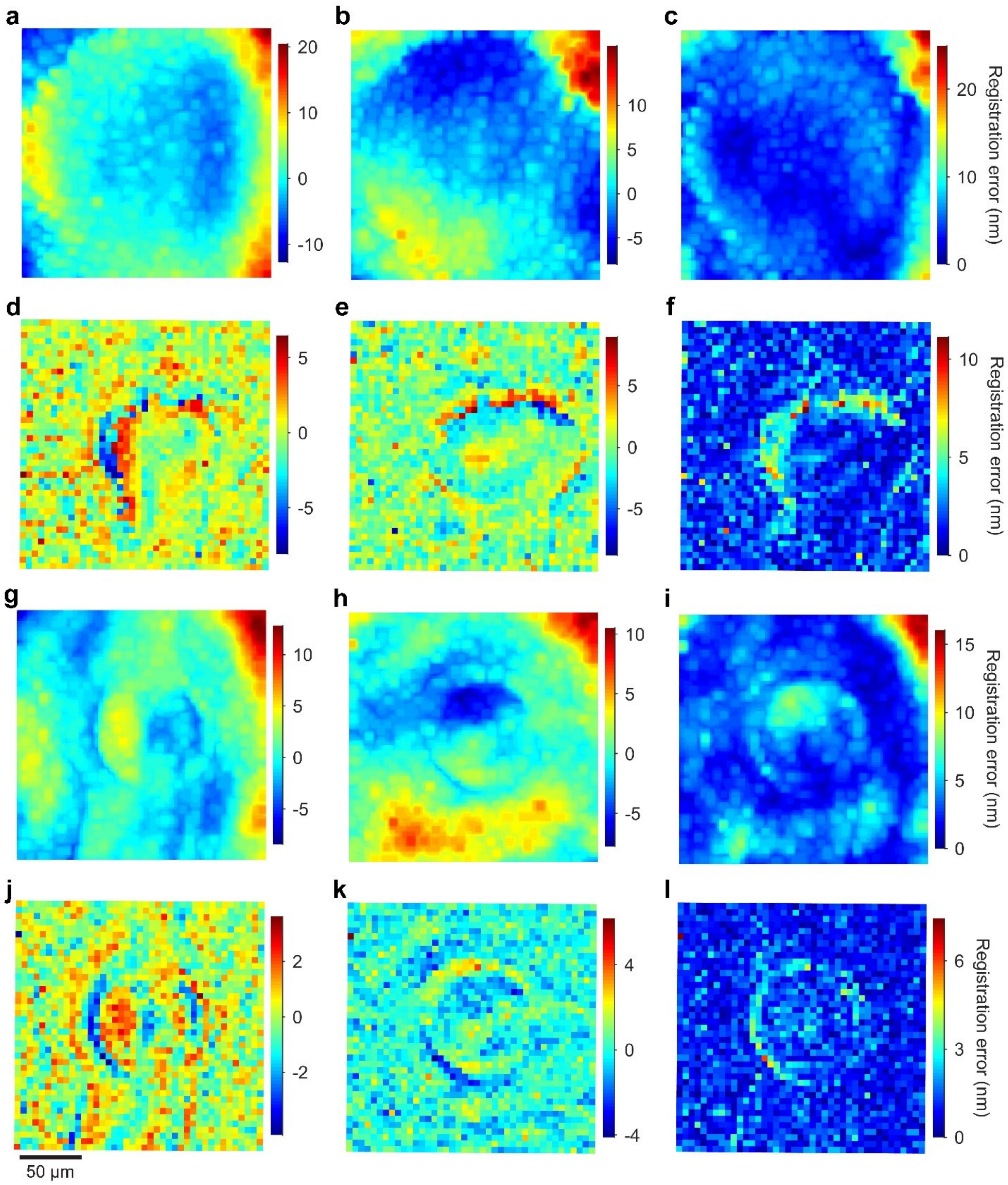

Figure S15. Registration errors from three colors at one focal plane. (**a-f**) Plots showing registration errors in (a,d) the x direction, (b,e) the y direction, and (c,f) total magnitude, (a-c) before correction and (d-f) after correction of data from 500 nm and 630 nm peak wavelengths, at the optimal focal plane for the former. (**g-l**) Plots showing registration errors in (g,j) the x direction, (h,k) the y direction, and (i,l) total magnitude (g-i) before correction and (j-l) after correction of data from 400 nm and 500 nm peak wavelengths, at the optimal focal plane for the former.

Systematic errors due to the wavelength dependence of distortion are apparent in the data before correction (a-f, h-j). Systematic errors due to defocus are apparent in the (a-f) 630 nm data and (h-m) 400 nm data.

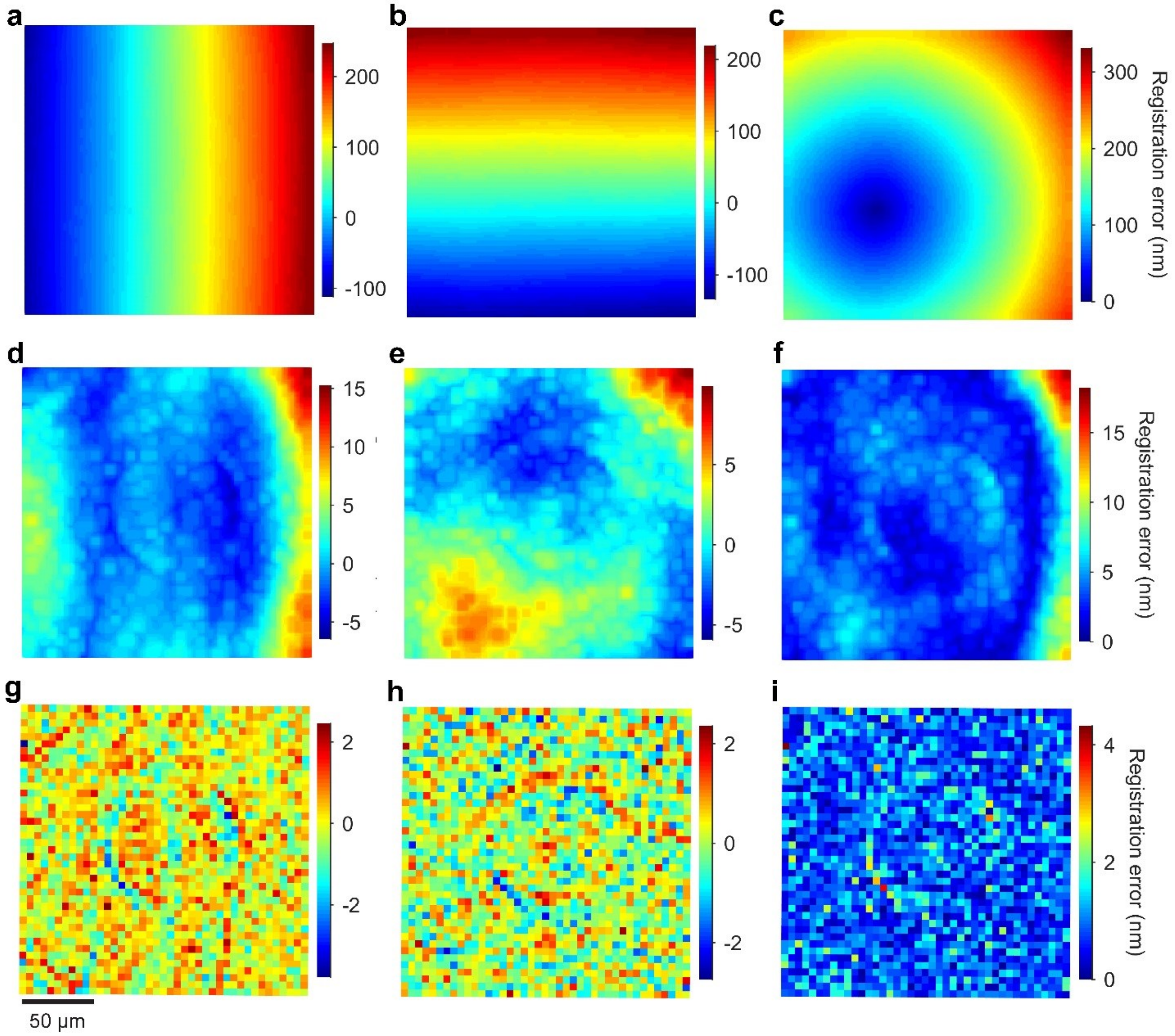

Figure S16. Registration errors from two colors at optimal focal planes. **(a-c)** Plots showing registration errors in (a) the x direction, (b) the y direction, and (c) total magnitude, due mostly to different mean values of image pixel size and a lateral offset for localization data from 400 nm and 500 nm peak wavelengths. **(d-f)** Plots showing registration errors in (d) the x direction, (e) the y direction, and (f) total magnitude, after applying a similarity transform to the localization data, due mostly to variable distortion from chromatic aberration. **(g-i)** Plots showing registration errors in (g) the x direction, (h) the y direction, and (i) total magnitude, after applying correction models to the localization data before a similarity transform, due mostly to localization error and empirical localization precision.

Note S9. Error analysis for multicolor registration

Registration errors of data after correction from two colors are due to a combination of empirical localization precision and localization error, having a variance of

$\sigma_{reg}^2 = \sigma_{lp,1}^2 + \sigma_{lp,2}^2 + \sigma_{le,1}^2 + \sigma_{le,2}^2$, (Eq. S12)

where $\sigma_{lp,1}^2$ and $\sigma_{lp,2}^2$ are the variance due to empirical localization precision, and $\sigma_{le,1}^2$ and $\sigma_{le,2}^2$ are the variance due to localization error for colors 1 and 2, respectively. Assuming the localization error is the same for each color channel, or equivalently considering the mean value, and by measuring the empirical localization precision, we determine the contribution of localization error to the registration error as

$\sigma_{le} = \sqrt{\frac{\sigma_{reg}^2 - \sigma_{lp,1}^2 - \sigma_{lp,2}^2}{2}}$. (Eq. S13)

Values of empirical localization precision are in Table S9. Values of the contribution of localization error to registration error, σ_{le}, for data before and after correction prior to registration are in Table S10.

Table S9. Empirical localization precision in multicolor registration

**Peak wavelength (nm)**	$\boldsymbol{\sigma_{lp,x}}$ **(nm)**	$\boldsymbol{\sigma_{lp,y}}$ **(nm)**
400	0.340 ± 0.003	0.318 ± 0.002
500	0.371 ± 0.003	0.315 ± 0.002
630	0.394 ± 0.002	0.320 ± 0.002

Uncertainties are one standard error.

Table S10. Localization error in multicolor registration

		**400 nm and 500 nm**		**500 nm and 630 nm**	
		$\boldsymbol{\sigma_{le,x}}$ **(nm)**	$\boldsymbol{\sigma_{le,y}}$ **(nm)**	$\boldsymbol{\sigma_{le,x}}$ **(nm)**	$\boldsymbol{\sigma_{le,y}}$ **(nm)**
Optimal focal planes	Uncorrected	2.23 ± 0.04	1.70 ± 0.03	2.45 ± 0.04	1.78 ± 0.03
	Corrected	0.40 ± 0.01	0.41 ± 0.01	0.35 ± 0.01	0.47 ± 0.01
Single focal plane	Uncorrected	1.85 ± 0.03	1.85 ± 0.03	2.86 ± 0.05	2.86 ± 0.05
	Corrected	0.63 ± 0.01	0.59 ± 0.01	1.16 ± 0.02	1.28 ± 0.02

Uncertainties are one standard error.

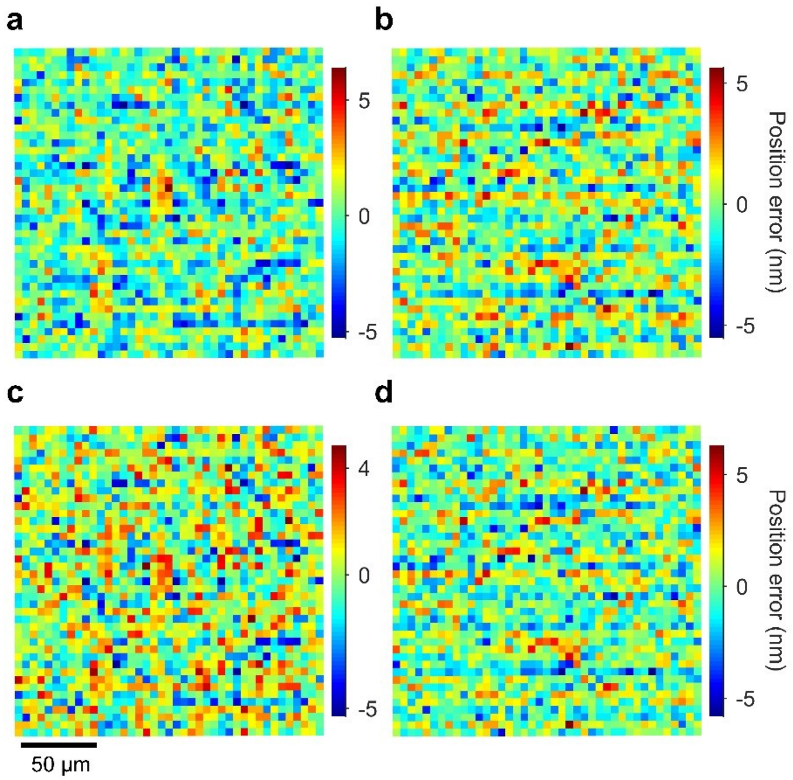

Figure S17. Correction of fluorescence data. (**a-d**) Plots showing position errors in (a,c) the x direction and (b,d) the y direction following correction of data from (a-b) transillumination and (c-d) fluorescence. These results show that our reference materials and calibration methods are equally applicable to transillumination of empty apertures and epiillumination of fluorescent dye in apertures.

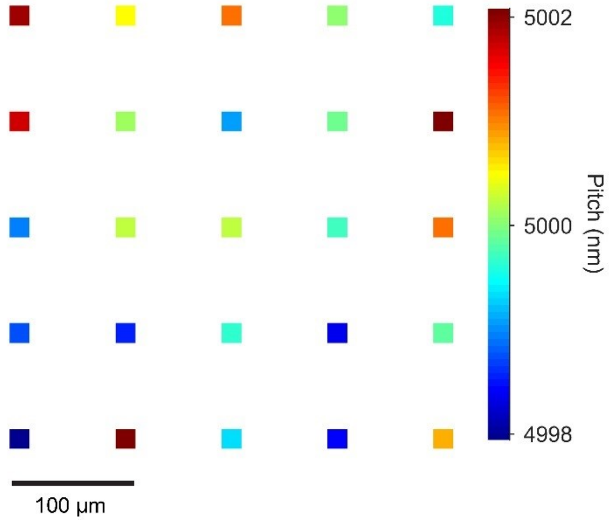

Figure S18. Pitch across the aperture array. Plot showing 25 regions of the aperture array, with color scale indicating the mean pitch from four aperture pairs within each region. Data marker size is not to scale. No systematic effects indicative of electron-optical aberrations are evident.

Table S11. Pitch characterization for two lithography systems

	**x direction**		**y direction**	
	**Array 1**	**Array 2**	**Array 1**	**Array 2**
Mean pitch (pixels)	49.969	49.958	49.974	49.964
Standard error (pixels)	0.003	0.002	0.002	0.002

Note S10. Rigidity analysis

The positions of each aperture or nanoparticle define a nominally rigid constellation of points in the image plane, $(x_{j,\eta}, y_{j,\eta})$, where the index j denotes an image in a measurement series and the index η denotes a point in a constellation[5]. We measure and remove the common-mode motion of the sample by applying a two-dimensional rigid transformation to map the constellation in image j to the constellation in image k. This transformation consists of a displacement of the centroid of the constellation $(X_j - X_k)\,\hat{x} + (Y_j - Y_k)\,\hat{y}$ and a rotation of the constellation about the centroid, $\Delta\theta = \theta_j - \theta_k$, where (X_j, Y_j) and (X_k, Y_k) are the positions of the centroids in images j and k, respectively, and θ_j and θ_k are the orientations of the constellation in images j and k, respectively. The optimal rigid transformation minimizes the registration error between corresponding points in images j and k. Registration error is insensitive to systematic errors in localizing single apertures or nanoparticles. Therefore, we omit CMOS calibration from this analysis.

Motion of a sample in the z direction during a time series can cause apparent deformation of a rigid constellation in optical micrographs. At time scales that allow, we minimize these effects by imaging through focus at each point in the time series, acquiring images at multiple z positions around the plane of optimal focus for the entire time series. The nominal spacing in z position between each image is 10 nm, set by the resolution of our piezoelectric nosepiece that controls the position of the objective lens. At each time point, we choose from the set of images at varying z positions the one image that minimizes the root-mean-square of the registration errors from registration with the first image in the time series. This procedure minimizes any motion of the sample in the z direction relative to the position at the initial time point, so that the images that form the resulting time series share a common z position within 10 nm.

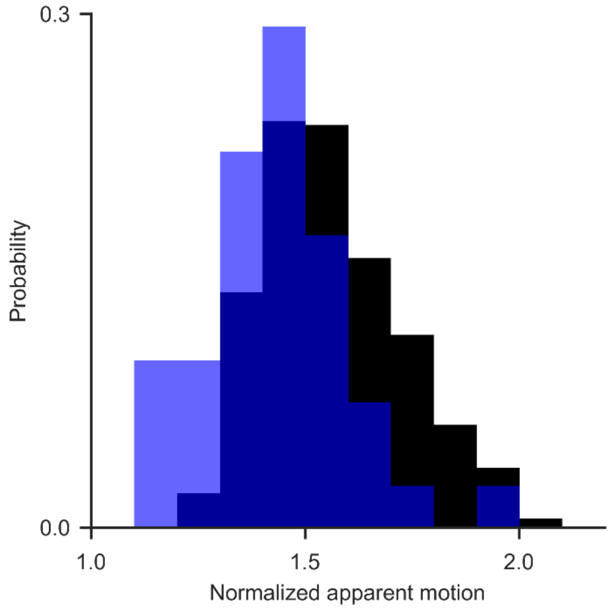

Figure S19. Nanoparticle stability down to 10^{-1} s. Plot showing probability distributions of normalized apparent motion for nominally motionless apertures (black) and nanoparticles (blue) that we image at a frequency of 10^{1} s^{-1} for a duration of 10^{1} s, without intentionally changing the z position. The normalization is with respect to the Cramér–Rao lower bound and accounts primarily for differences in the number of signal photons. The corresponding absolute mean values define the measurement uncertainties, and are approximately 0.43 nm for apertures and 0.55 nm for nanoparticles. The magnitude of normalized apparent motion for nanoparticles is comparable to that of static apertures, indicating that the nanoparticles are also static at these scales.

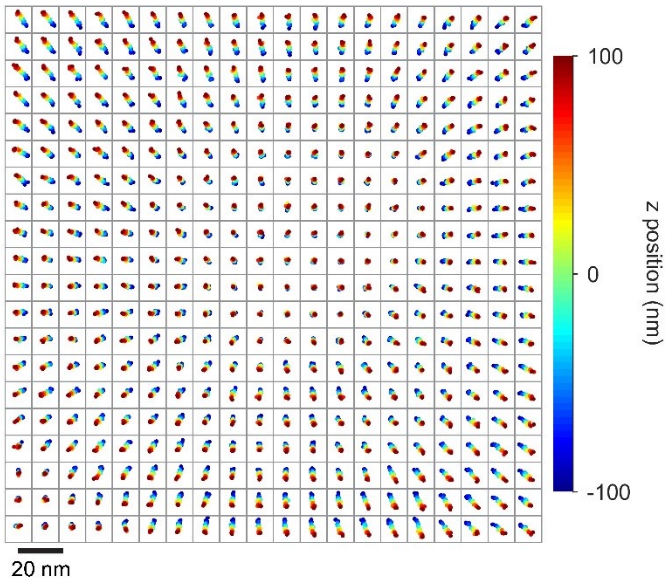

Figure S20. Apparent motion. Grid of scatterplots, each corresponding to a single aperture, showing apparent motion in the radial direction due to imaging through focus over a range of 200 nm in z position. The grid spacing indicates an array pitch of 10 µm. The scale bar corresponds to the scatterplots.

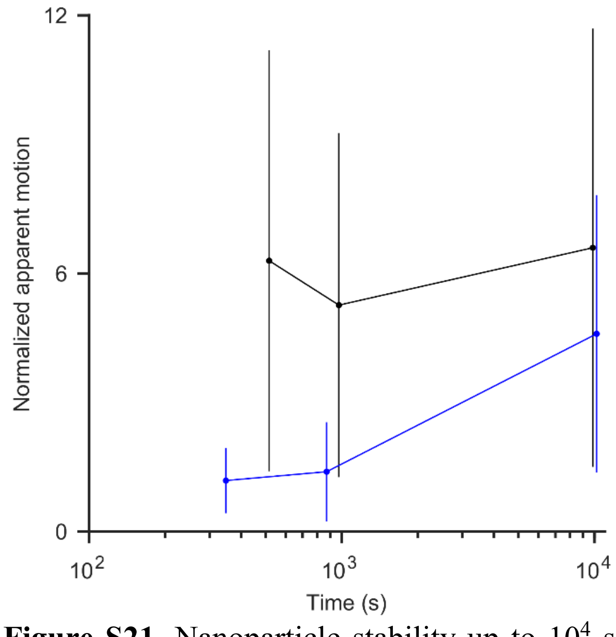

Figure S21. Nanoparticle stability up to 10^4 s. Plot showing normalized apparent motion as a function of time, exceeding the time that is necessary for imaging through focus, for nominally static apertures (black) and nanoparticles (blue). Normalization is with respect to empirical localization precision, or the corresponding values of apparent motion at the time scale of 10^{-1} s. Data markers are mean values and vertical bars are ± one standard deviation. The values of normalized apparent motion for nanoparticles are comparable to those of apertures, indicating that the nanoparticles are static at these scales.

References

1 Zernike VF. Beugungstheorie des schneidenver-fahrens und seiner verbesserten form, der phasenkontrastmethode. *Physica* 1934; **1**: 689-704.

2 Mortensen KI, Churchman LS, Spudich JA, Flyvbjerg H. Optimized localization analysis for single-molecule tracking and super-resolution microscopy. *Nat Methods* 2010; **7**: 377-381.

3 Abraham AV, Ram S, Chao J, Ward ES, Ober RJ. Quantitative study of single molecule location estimation techniques. *Opt Express* 2009; **17**: 23352-23373.

4 Huang F, Hartwich TMP, Rivera-Molina FE, Lin Y, Duim WC *et al.* Video-rate nanoscopy using sCMOS camera-specific single-molecule localization algorithms. *Nat Methods* 2013; **10**: 653-658.

5 McGray C, Copeland CR, Stavis SM, Geist J. Centroid precision and orientation precision of planar localization microscopy. *J Microsc* 2016; **263**: 238-249.
